# Progesterone receptor membrane component 1 leads to erlotinib resistance, initiating crosstalk of Wnt/β-catenin and NF-κB pathways, in lung adenocarcinoma cells

**DOI:** 10.1038/s41598-020-61727-3

**Published:** 2020-03-16

**Authors:** Ying Lin, Kazuma Higashisaka, Takuya Shintani, Ayaka Maki, Sachiyo Hanamuro, Yuya Haga, Shinichiro Maeda, Hirofumi Tsujino, Kazuya Nagano, Yasushi Fujio, Yasuo Tsutsumi

**Affiliations:** 1 Laboratory of Toxicology and Safety Science, Graduate School of Pharmaceutical Sciences, Osaka University, 1-6 Yamadaoka, Suita, Osaka 565-0871 Japan; 2 Department of Legal Medicine, Osaka University Graduate School of Medicine, 2-2 Yamadaoka, Suita, Osaka 565-0871 Japan; 3 Department of Pharmacy, Osaka University Hospital, 2-15 Yamadaoka, Suita, Osaka 565-0871 Japan; 4 Advanced Research of Medical and Pharmaceutical Sciences, Graduate School of Pharmaceutical Sciences, Osaka University, 1-6 Yamadaoka, Suita, Osaka 565-0871 Japan; 5 Laboratory of Clinical Science and Biomedicine, Graduate School of Pharmaceutical Sciences, Osaka University, 1-6 Yamadaoka, Suita, Osaka 565-0871 Japan; 6 Global Center for Medical Engineering and Informatics, Osaka University, 2-2 Yamadaoka, Suita, Osaka 565-0871 Japan

**Keywords:** Non-small-cell lung cancer, Mechanisms of disease

## Abstract

In non-small-cell lung cancer, mutation of epidermal growth factor receptor (EGFR) stimulates cell proliferation and survival. EGFR tyrosine kinase inhibitors (EGFR-TKIs) such as erlotinib are used as first-line therapy with drastic and immediate effectiveness. However, the disease eventually progresses in most cases within a few years due to the development of drug resistance. Here, we explored the role of progesterone membrane component 1 (PGRMC1) in acquired resistance to erlotinib and addressed the molecular mechanism of EGFR-TKI resistance induced by PGRMC1. The erlotinib-sensitive cell line PC9 (derived from non-small-cell lung cancer) and the erlotinib-resistant cell line PC9/ER were used. In proteomic and immunoblotting analyses, the PGRMC1 level was higher in PC9/ER cells than in PC9 cells. WST-8 assay revealed that inhibition of PGRMC1 by siRNA or AG-205, which alters the spectroscopic properties of the PGRMC1-heme complex, in PC9/ER cells increased the sensitivity to erlotinib, and overexpression of PGRMC1 in PC9 cells reduced their susceptibility to erlotinib. In the presence of erlotinib, immunoprecipitation assay showed that AG-205 suppressed the interaction between EGFR and PGRMC1 in PC9/ER cells. AG-205 decreased the expression of β-catenin, accompanied by up-regulation of IκBα (also known as NFKBIA). Furthermore, AG-205 reduced the expression of β-TrCP (also known as BTRC), suggesting that PGRMC1 enhanced the crosstalk between NF-κB (also known as NFKB) signaling and Wnt/β-catenin signaling in an erlotinib-dependent manner. Finally, treatment with the Wnt/β-catenin inhibitor XAV939 enhanced the sensitivity of PC9/ER cells to erlotinib. These results suggest that PGRMC1 conferred resistance to erlotinib through binding with EGFR in PC9/ER cells, initiating crosstalk between the Wnt/β-catenin and NF-κB pathways.

## Introduction

Lung cancer, the leading cause of cancer death worldwide, can be classified into small-cell lung cancer (SCLC) and non-small-cell lung cancer (NSCLC). Patients diagnosed with NSCLC account for 85% of total lung cancer^1^, and have a five-year survival rate of less than 17%^2^. Unlike in SCLC, the conventional therapies such as chemotherapy and radiotherapy generally have a weak effect on NSCLC, especially when the tumor has been proliferated to advanced stage. In NSCLC, epidermal growth factor receptor (EGFR), stimulates cell proliferation and survival^3^. Aberrations of EGFR (about 62.2% of which are a deletion in exon 19) drive NSCLC by activating MAP kinase or PI3K/Akt signaling^4^. Thus, in clinical practice, EGFR tyrosine-kinase inhibitors (EGFR-TKIs) have a dramatic effect on NSCLC patients carrying an EGFR mutation. Since the first generation of EGFR-TKIs (e.g., gefitinib and erlotinib) were approved by the US Food and Drug Administration for clinical use in 2003, there has been a striking increase in median progression-free survival^5^. Consequently, EGFR-TKI treatment has become the optimal choice for treating NSCLC patients with an EGFR mutation.

However, in NSCLC patients treated with one or more EGFR-TKIs, drug resistance emerges within about one year of continuous therapy in almost all cases followed by disease progression, acquired resistance develops and limits the long-term efficacy of these EGFR TKIs, leading to a lower quality of life^6,7^. Thus, the mechanism of EGFR-TKI resistance has been a focus of clinical research. Three causes of resistance are commonly reported^8^: (1) The most reported cause is a T790M mutation in EGFR, which attenuates the binding of EGFR-TKIs to EGFR^9^; (2) Amplification of receptors such as MET and HER2 (also known as ERBB2) can activate downstream pathways like PI3K/Akt signaling axis compensating the inhibitory effect of EGFR-TKIs on EGFR^7,10^; (3) Histologic transformation to SCLCs occurs in NSCLCs patients after an initial response to EGFR-TKIs and results in less sensitivity to EGFR-TKIs^11,12^. There remains an immediate need to further elucidate the mechanism of EGFR-TKI resistance and establish an effective solution to this problem.

By performing proteome analysis with a newly established erlotinib-resistant cell line, PC9/ER, derived from PC9 lung cancer cells^13^, we previously revealed progesterone membrane component 1 (PGRMC1) as a candidate protein involved in acquired resistance to erlotinib. Recently, PGRMC1 was reported to be overexpressed in breast cancer^14^, lung cancer^15^, and ovarian cancer^16^. Heme-dependent dimerization of PGRMC1 has been shown to accelerate tumor growth through the EGFR signaling pathway and facilitate cancer proliferation and chemoresistance^17^. PGRMC1 also plays a key role in the development of gefitinib-associated resistance^18^. However, the detailed signaling pathway mechanism by which PGRMC1 promotes acquired resistance to EGFR-TKIs is still obscure.

Here, we demonstrated that PGRMC1 inhibition leads to enhanced susceptibility to erlotinib resistance. These results suggest that PGRMC1 attenuates the EGFR-TKI efficiency via activation of Wnt/β-catenin and NF-κB pathways in NSCLC and that PGRMC1 is a potential target molecular to overcome EGFR-TKI resistance.

## Methods

### Cell lines and cell cultures

The NSCLC cell line PC9 (erlotinib-sensitive; EGFR exon 19-bp deletion from E746 to A750) was purchased from the RIKEN BioResource Center (Tsukuba, Japan). The erlotinib-resistant cell line PC9/ER was obtained from the PC9 cell line by exposing the cells to increasing concentrations of erlotinib for 6 months. The PC9 cell line was cultured in RPMI-1640 (Wako, Osaka, Japan) with 10% (v/v) fetal bovine serum (FBS; Gibco, Waltham, MA, USA) and 1% (v/v) Antibiotic-Antimycotic (100X) (Ab; Thermo Fisher Scientific, Waltham, MA, USA) and maintained at 37 °C with 5% CO_2_ and over 95% humidity. The PC9/ER cell line was cultured in the same conditions as the PC9 cell line with the addition of 5 μM erlotinib (Selleckchem, Houston, TX, USA).

### Cell viability

PC9 and PC9/ER cells were seeded at 8000 cells/well in 96-well flat plates overnight without drugs; the medium was then replaced and the cells were treated with erlotinib (20 nM) alone or erlotinib plus various concentrations of AG-205 (TimTec, Newark, DE, USA), XAV939, JSH-23, or MG-132 (Sigma-Aldrich, Darmstadt, DE, USA), or GS-143 (R&D Systems, Inc., Minneapolis, MN, USA) for 72 h, and cell viability was evaluated by WST-8 assay (Nacalai Tesque, Inc., Kyoto, Japan). Erlotinib, AG-205, XAV939, JSH-23, MG-132, and GS-143 were dissolved in dimethylsulfoxide (DMSO) and stored at −20 °C prior to use. Cell viability following transfection with the EGFR-expressing plasmid and *PGRMC1* siRNAs and control siRNAs described below were also assayed by WST-8 assay.

### Immunoblotting analysis

Proteins were extracted by M-PER Mammalian Protein Extraction Reagent with Protease Inhibitor Cocktail (both from Thermo Fisher Scientific). Proteins were mixed with an equal volume of Laemmli sample buffer (Bio-Rad Laboratories, Hercules, CA, USA) containing 5% (v/v) 2-mercaptoethanol and then boiled for 5 min prior to separation by sodium dodecyl sulfate polyacrylamide gel electrophoresis. Precision Plus Protein Kaleidoscope molecular weight markers (Bio-Rad laboratories) were used as standards. The proteins were then electro-transferred onto a polyvinylidene difluoride membrane (Millipore, Bedford, MA, USA) and blocked with 4% (w/v) Block Ace (KAC Co., Ltd., Kyoto, Japan) in phosphate buffered saline plus 0.01% (v/v) Triton X-100 (PBST). The membranes were incubated with primary antibody for 1 h and then treated with secondary antibody for 1 h. The following antibodies were used: anti-PGRMC1 monoclonal antibody (mAb) (#13856, 1:1000), anti-β-TrCP mAb (#4394, 1:200), anti-IκBα mAb (#4812, 1:1000), and anti-rabbit IgG-horseradish peroxidase-conjugated secondary antibody (#7074 S, 1:2000) (Cell Signaling Technology, Danvers, MA, USA); anti-β-catenin mAb (sc-7963, 1:1000); anti-β-actin mAb (A5316, 1:50000) and anti-mouse IgG secondary antibody (Sigma-Aldrich). All antibodies were diluted in 0.4% (w/v) Block Ace in PBST. The protein bands were detected by using SuperSignalWest Femto Maximum Sensitivity Substrate (Thermo Fisher Scientific) and visualized with an ImageQuant LAS 4000 mini biomolecular imager (GE Healthcare Japan, Tokyo, Japan). The resultant images were analyzed by using ImageJ JAVA 1.6.0_24 (64-bit) (National Institutes of Health, Bethesda, MD, USA).

### Transient transfection of negative control and/or PGRMC1 small interfering RNA (NC and/or PGRMC1 siRNA)

Cells were transfected with 50 nM Stealth siRNA against *PGRMC1* (5′-GGGAGUCUCAGUUCAUUUtt-3′ and 3′AAAGUGAACUGACUCCCag-5′) or Stealth siRNA negative control with medium GC content (Invitrogen, Carlsbad, CA, USA). The transfection was executed with Lipofectamine RNAiMAX Transfection Reagent (Invitrogen) for 72 h in RPMI-1640 containing 10% FBS and 1% Ab.

### Transfection of PGRMC1 vector

pCMV3 plasmid encoding human PGRMC1 was purchased from Sino Biological Inc. (Beijing, China). The insert cDNA contained the complete PGRMC1 coding sequence (NM_006667.4). The pCMV3-PGRMC1 and pCMV3 negative control vectors were separately transfected into PC9 cells at 70–80% confluency using Lipofectamine 2000 Transfection Reagent (Invitrogen) for 72 h in RPMI-1640 containing 10% FBS.

### Immunoprecipitation

Proteins were extracted as described above for immunoblotting analysis, and protein concentrations were evaluated by bicinchoninic acid (BCA) assay (Thermo Fisher Scientific). Protein A Mag Sepharose (GE Healthcare) was transferred to clean tubes and washed using a Protein A/G HP SpinTrap Buffer Kit and magnetic separation rack (GE Healthcare). After adding anti-EGFR, the samples were incubated with rotation for 1 h at room temperature, and the beads were separated from the lysate. Protein diluted with binding buffer was mixed with the beads, and the suspension was incubated with rotation at room temperature for 1 h. Then, elution buffer was added to the samples and the elution was collected.

### Statistical analysis

**Figure 1:**
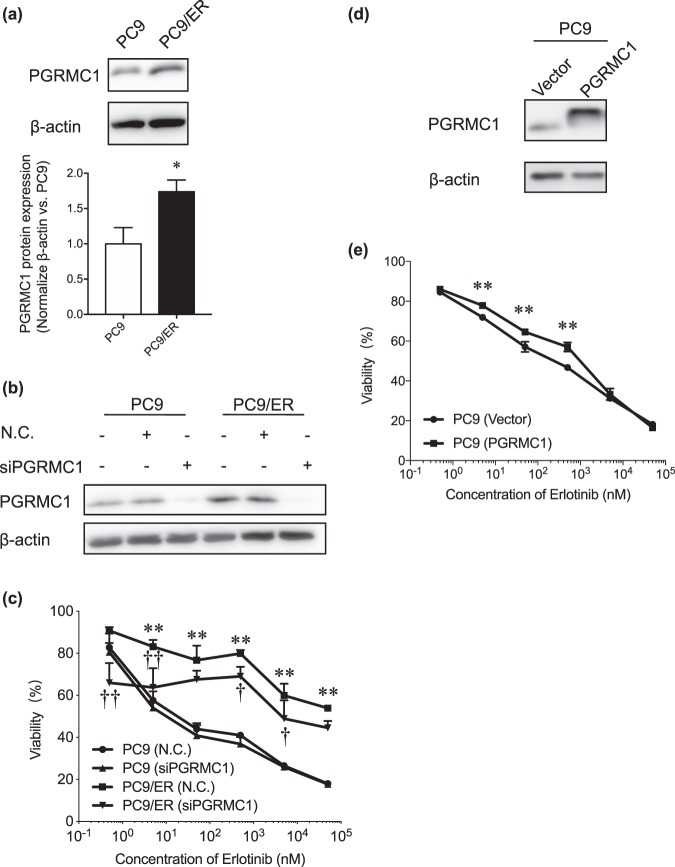
Contribution of PGRMC1 to the resistance to erlotinib in lung adenocarcinomas cancer. (**a**) Immunoblotting of PGRMC1 in PC9 and PC9/ER cells. The upper panel shows a representative blot. The lower panel shows means ± SD (n = 3); *P < 0.05 vs. PC9 (two-tailed Student *t*-test). (**b**) Immunoblotting analysis of the efficiency of transfection of PC9 and PC9/ER cells with *PGRMC1* siRNA (siPGRMC1). NC, negative control. The results shown are representative of 2 independent experiments. (**c**) WST-8 assay of the effects of various concentrations of erlotinib on cell viability of *PGRMC1* siRNA–treated PC9 and PC9/ER cells. Data are means ± SD (n = 3); **P < 0.01, *P < 0.05 vs. PC9 (negative control); ^†^*P* < 0.05, ^††^*P* < 0.01 vs. PC9/ER (negative control) (Two-Way ANOVA followed by Tukey correction). NC, negative control. (**d**) Immunoblotting analysis of the efficiency of transfection of PC9 cells with a PGRMC1-expressing plasmid. (**e**) WST-8 assay of the effects of various concentrations of erlotinib on PC9 cells transfected with empty vector or pCMV3-PGRMC1. Data are means ± SD (n = 3); **P < 0.01 vs. PC9 with empty vector (Two-Way ANOVA followed by Bonferroni correction).

**Figure 2:**
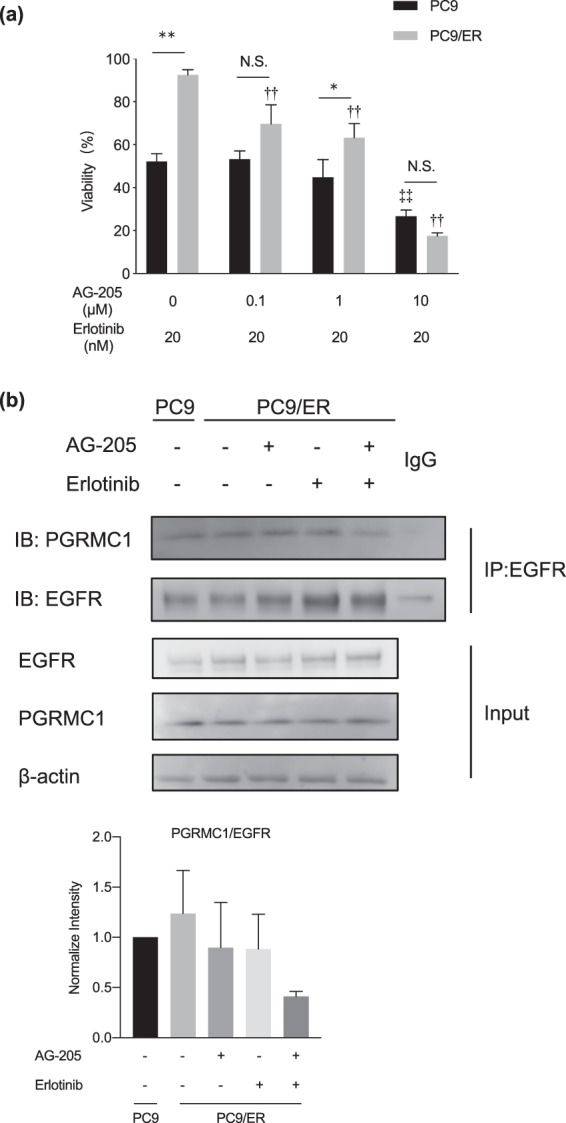
Effects of combination treatment of erlotinib and AG-205 on cell viability and EGFR–PGRMC1 binding in lung adenocarcinoma cells. (**a**) Effects of combination treatment of erlotinib and AG-205 on cell viability measured by WST-8 assay. Data are means ± SD (n = 3); **P < 0.01 PC9 vs. PC9/ER, ^††^*P* < 0.01 *vs*. *P*C9/ER cells treated with erlotinib alone, ^‡‡^*P* < 0.01 vs. PC9 cells treated with erlotinib alone (Two-Way ANOVA followed by Bonferroni correction); N.S; not significant. (**b**) Immunoprecipitation assay of binding between EGFR and PGRMC1 in PC9 and PC9/ER cells cotreated with AG-205 (1 μM) plus erlotinib (20 nM) for 72 h. Data are means ± SD (n = 3).

**Figure 3:**
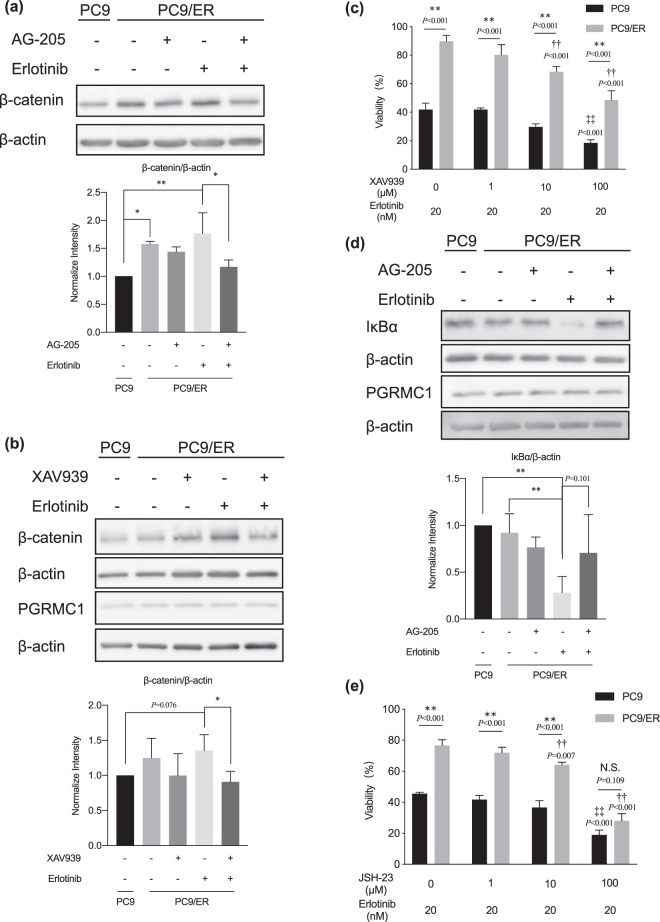
Effects of combination treatment of erlotinib and XAV939/JSH-23 on erlotinib resistance. (**a**,**b**) Immunoblotting analysis of β-catenin expression in cells cotreated with erlotinib (20 nM) plus (**a**) AG-205 (1 μM) or (**b**) XAV939 (10 μM) for 72 h. Data are means ± SD (n = 6); *P < 0.05, **P < 0.01 (One-Way ANOVA followed by Tukey correction). (**c**) WST-8 assay of the effects of combination treatment of XAV939 plus erlotinib on cell viability. Data are means ± SD (n = 6); **P < 0.01 PC9 vs. PC9/ER, ^††^*P* < 0.01 vs. PC9/ER cells treated with erlotinib alone, ^‡‡^*P* < 0.01 vs. PC9 cells treated with erlotinib alone (Two-Way ANOVA followed by Bonferroni correction). (**d**) Immunoblotting analysis of IκBα expression in cells cotreated with AG-205 (1 μM) plus erlotinib (20 nM) for 30 min. Data are means ± SD (n = 4); **P < 0.01 (One-Way ANOVA followed by Tukey correction). (**e**) WST-8 assay of the effects of combination treatment of JSH-23 plus erlotinib on cell viability. Data are means ± SD (n = 3); **P < 0.01 PC9 vs. PC9/ER, ^††^*P* < 0.01 vs. PC9/ER cells treated with erlotinib alone, ^‡‡^*P* < 0.01 vs. PC9 cells treated with erlotinib alone (Two-Way ANOVA followed by Bonferroni correction); N.S; not significant.

**Figure 4:**
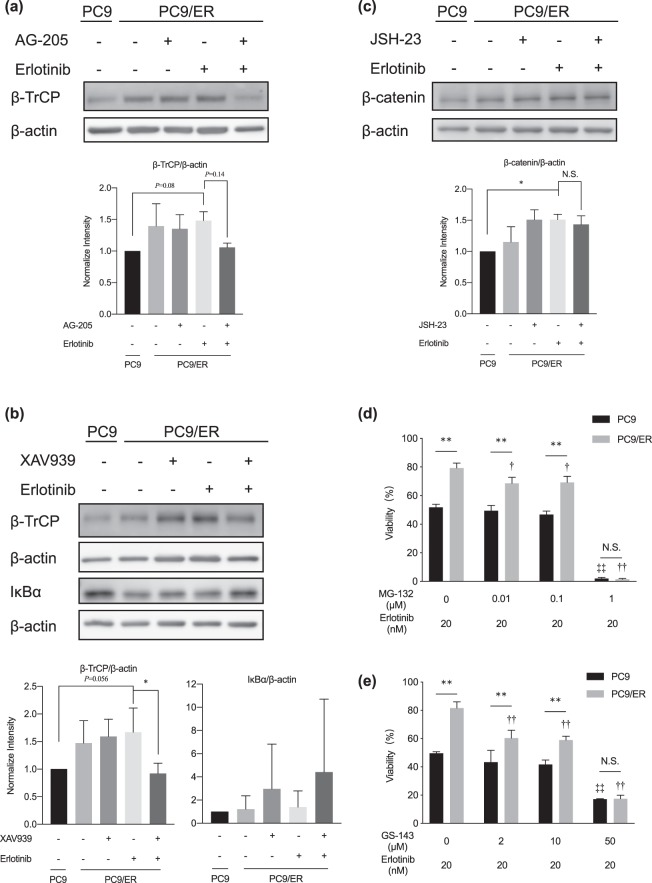
Crosstalk between the Wnt/β-catenin and NF-κB pathways. (**a**) Immunoblotting analysis of the expression of β-TrCP in PC9 and PC9/ER cells cotreated with AG-205 (1 μM) plus erlotinib (20 nM). Data are means ± SD (n = 3); (One-Way ANOVA followed by Tukey correction). (**b**) Immunoblotting analysis of the expression levels of β-TrCP and IκBα in cells cotreated with XAV939 (10 μM) plus erlotinib (20 nM) for 72 h and 30 min, respectively. Data are means ± SD (n = 3); *P < 0.05 (One-Way ANOVA followed by Tukey correction). (**c**) Immunoblotting analysis of the expression of β-catenin in cells cotreated with JSH-23 (10 μM) and erlotinib (20 nM). Data are means ± SD (n = 3); *P < 0.05 (One-Way ANOVA followed by Tukey correction). N.S; not significant. (**d**,**e**) WST-8 assay of the effects of combination treatment of erlotinib and (**d**) MG-132 or (**e**) GS-143 on cell viability. Data are means ± SD (n = 3); **P < 0.01, ^†^*P* < 0.05, ^††^*P* < 0.01 vs. PC9/ER cells treated with erlotinib alone, ^‡‡^*P* < 0.01 vs. PC9 cells treated with erlotinib alone (Two-Way ANOVA followed by Bonferroni correction); N.S; not significant.

All statistical analyses were conducted using Graph Pad Prism Mac version 7.0 (GraphPad Software, La Jolla, CA; www.graphpad.com). Data are expressed as means ± SD. For the results in Fig. 1a the two-sided Student’s *t*-test method was used to compare groups. For the results in Figs. 1c,e, 2a, 3c,e and 4d,e, Two-Way ANOVA followed by Bonferroni correction was used to compare groups.

## Results

### PGRMC1 contributes to acquired resistance to erlotinib

To elucidate the molecular mechanism of acquired EGFR-TKI resistance, we previously compared PC9 and PC9/ER cells by proteomics analysis^13^; the results indicated that PGRMC1 expression in PC9/ER cells was 3.8-fold that in PC9 cells (Table 1). To validate the clinical relevance of PGRMC1, Kaplan-Meier plots regarding PGRMC1 expression and overall survival in the lung cancer revealed that high expression of PGRMC1 patients have less overall survival rate than low expression of PGRMC1 patients (Additional file 1: Fig. S1a). In addition, we checked genomic alternation of PGRMC1 in NSCLC subtypes and OncoPrint outputs found that gene alterations of PGRMC1 was in 1.84% of 1,144 cases (Additional file 1: Fig. S1b). The information suggests that the expression level or genomic alternation of PGRMC1 has clinical relevance in lung cancer.

**Table 1:** Proteomics result for PGRMC1 levels in PC9 and PC9/ER cells.

Cells	Unique peptide	Peptide-spectrum matches	Score	Peak area
PC9	5	7	106.17	2.557E6
PC9/ER	8	12	251.35	9.808E6

Here, by conducting immunoblotting, we confirmed that the protein level of PGRMC1 was up-regulated in PC9/ER cells compared with the parental cell line (Fig. 1a). Initially, to evaluate the role of PGRMC1 in acquired resistance to erlotinib, we used the WST-8 assay to examine sensitivity to erlotinib under conditions with or without PGRMC1 siRNA in the two cell lines; immunoblotting confirmed the efficient knockdown of PGRMC1 by the siRNA (Fig. 1b). Both cell lines showed dose-dependency of erlotinib-induced loss of viability; PC9/ER cells were more resistant than PC9 cells (Fig. 1c). Following erlotinib treatment, the viability of PC9 cells transfected with *PGRMC1* siRNA was similar to that of those transfected with the siRNA negative control; in contrast, PC9/ER cells transfected with *PGRMC1* siRNA displayed lower viability than those transfected with the siRNA negative control, especially at low concentrations of erlotinib (0.5–500 nM). Moreover, PC9 cells transfected with pCMV3-PGRMC1 to provide overexpression of PGRMC1 also showed up-regulation of PGRMC1 expression in PC9 cells by immunoblotting (Fig. 1d) and significant attenuation of susceptibility to erlotinib by WST-8 assay (Fig. 1e). These results suggest that PGRMC1 contributes to the acquired erlotinib resistance in PC9 cells.

### Enhanced binding between PGRMC1 and EGFR in PC9/ER cells

We then compared the effect of the PGRMC1 inhibitor AG-205, which alters the spectroscopic properties of the heme-PGRMC1 complex^19^, on erlotinib sensitivity in the two cell lines. In the subsequent studies, we used the concentration erlotinib (20 nM), at which the effect of siPGRMC1 was remarkably observed (Fig. 1c). The viability of PC9 cells treated with AG-205 (0.1, 1, or 10 μM) plus erlotinib (20 nM) was similar to that of those treated with erlotinib alone; in contrast, the viability of PC9/ER cells was significantly less following the combined treatment compared with erlotinib alone (Fig. 2a). Because the heme-PGRMC1 complex binds with EGFR to activate EGFR downstream signaling pathways^17^, we supposed that the binding between PGRMC1 and EGFR might be responsible for the reduction of the viability of PC9/ER cells following combination treatment with AG-205 plus erlotinib. To test this hypothesis, we performed immunoprecipitation of EGFR in the two cell lines following treatment with AG-205 (1 μM) and/or erlotinib (20 nM). In PC9 cells, although the immunoblotting of PGRMC1 showed little change in each group, erlotinib single treatment could reduce the binding ability of EGFR and PGRMC1, and the AG-205 further addition showed no fluctuation than erlotinib group (Additional file 1: Fig. S2). In the meantime, although the immunoblotting of PGRMC1 showed little change in each group, the results in PC9/ER cells revealed that the EGFR binding activity was suppressed in the PC9/ER cells treated with AG-205 plus erlotinib compared with those treated with erlotinib alone (Fig. 2b). These results showed that the difference of interaction between EGFR and PGRMC1 under erlotinib treatment indicated the resistance ability to erlotinib.

However, when we cotreated PC9/ER cells with succinylacetone (an inhibitor of heme biosynthesis; 1 or 10 mM) plus erlotinib, we found significant effect on cell viability compared with erlotinib alone in both PC9 and PC9/ER (Additional file 1: Fig. S3), which showed that heme-inhibitor have the similar effect in PC9/ER as in PC9. On the other hand, the immunoblotting analysis of three major phosphor-EGFR sites (at Tyr1068, Tyr992, and Tyr845), and phosphor-Akt and phosphor-ERK in PC9/ER cells showed less correlation with viability decrease, if any, for the AG-205 plus erlotinib group vs. erlotinib group (Additional file 1: Fig. S4). These results suggest that EGFR, Akt, and ERK activation have a limited role in the erlotinib resistance of PC9/ER cells.

### Activation of the Wnt/β-catenin and NF-κB pathways by interaction between PGRMC1 and EGFR in PC9/ER cells

PGRMC1 suppresses the Wnt/β-catenin pathway to promote human pluripotent stem cell self-renewal^20^; this in turn activates the NF-κB signaling pathway, which upregulates matrix metalloproteinase 9 gene expression and promotes tumor invasion^21^. Given that these pathways promote acquired EGFR-TKI resistance^22,23^, we hypothesized that interaction between PGRMC1 and EGFR activates the Wnt/β-catenin and NF-κB pathways in acquired erlotinib resistance in PC9 cells. Here, we observed that the level of β-catenin was induced significantly in PC9/ER cells when they were treated with erlotinib compared with non-treated group in PC9 cells (Fig. 3a). In contrast, although AG-205 showed little effect on the level of β-catenin, combination treatment with AG-205 and erlotinib (which showed no change of the expression of PGRMC1 but decreased the binding ability between EGFR and PGRMC1 in PC9/ER cells; Fig. 2b) downregulated the level of β-catenin significantly compared with the erlotinib treatment in PC9/ER cells. Moreover, the expression of nucleus β-catenin was also inhibited when treated AG-205 with erlotinib in PC9/ER cells (Additional file 1: Fig. S5a). To confirm the role of the Wnt/β-catenin pathway in erlotinib resistance, we evaluated the erlotinib sensitivity of PC9/ER cells after cotreatment with the β-catenin inhibitor XAV939 (10 μM) plus erlotinib. The expression of PGRMC1 showed no change even co-treated by XAV939 with erlotinib and the results showed that β-catenin activation was significantly downregulated in PC9/ER cells after cotreatment compared with cells treated with erlotinib alone (Fig. 3b, Additional file 1: Fig. S5b). In the meantime, compared to non-treat group, there was no significant change in the level of IκBα, β-catenin, or β-TrCP even treated with erlotinib. Also, the co-treatment group also showed little difference with erlotinib group in PC9 cells (Additional file 1: Fig. S6a). Furthermore, WST-8 assay showed that the cell viability in PC9/ER cells cotreated with 10 or 100 μM XAV939 plus erlotinib was significantly lower than in those treated with erlotinib alone (Fig. 3c); a similar trend was observed for 1 μM XAV939 plus erlotinib, but was not significant. The expression level of IκBα, which inhibits the nuclear translocation of NF-κB, was reduced in PC9/ER cells treated with erlotinib alone compared with the nontreatment control, but was enhanced in those treated with AG-205 plus erlotinib to reach a level similar to that in the nontreatment control (Fig. 3d). In the meantime, compared to non-treat group, there was no significant change in the level of IκBα, β-catenin, or β-TrCP even treated with erlotinib. Also, the co-treatment group also showed little difference with erlotinib group in PC9 cells (Additional file 1: Fig. S6b). In addition, the cell viability of the PC9/ER cells was significantly decreased after cotreatment with 10 or 100 μM JSH-23, which inhibit NF-κB nuclear translocation without effect on IκBα, plus erlotinib compared with treatment with erlotinib alone (Fig. 3e); a similar trend was observed for 1 μM JSH-23 plus erlotinib but this was not significant. These results suggest that binding of PGRMC1 to EGFR might activate Wnt/β-catenin and NF-κB pathways, leading to the resistance to erlotinib in PC9/ER cells.

### Crosstalk between Wnt/β-catenin and NF-κB pathways in PC9/ER cells

Recently, it has been reported that Wnt/β-catenin pathway is activating NF-κB pathway via β-TrCP^24^, which mediates ubiquitination and degradation of IκBα^25^. The mRNA of β-TrCP is stabilized when the Wnt/β-catenin signaling pathway is induced; the β-TrCP protein then degrades IκBα leading to induction of the NF-κB signaling pathway^26^. Here, comparing to PC9 cells, we found that the level of β-TrCP was induced in non-treated PC9/ER cells, and the treatment with erlotinib showed further up-regulation of β-TrCP. However, β-TrCP was observed to be reduced when PC9/ER cells were cotreated with AG-205 plus erlotinib rather than erlotinib alone (Fig. 4a), indicating that inhibition of PGRMC1 suppresses β-TrCP expression. Furthermore, compared to the erlotinib single treatment in PC9/ER cells, the expression of β-TrCP was reduced and the expression of IκBα was induced when the PC9/ER cells were cotreated with XAV939 plus erlotinib (Fig. 4b), suggesting that inhibiting the Wnt/β-catenin pathway suppressed the levels of β-TrCP thereby elevating the levels of IκBα. In contrast, β-catenin activation remained steady when PC9/ER cells were cotreated with JSH-23 plus erlotinib (Fig. 4c, Additional file 1: Fig. S5c), suggesting that inhibition of the NF-κB pathway had no effect on β-catenin levels. To inhibit the function of β-TrCP, which generally acts as a ubiquitin ligase leading to IκBα proteasomal degradation, in separate experiments we treated PC9/ER cells with proteasome inhibitor MG-132 plus erlotinib (Fig. 4d) or β-TrCP ligase inhibitor GS-143 plus erlotinib (Fig. 4e); in both of these experiments, the sensitivity of PC9/ER cells to erlotinib was enhanced by the combination treatment. On the other hand, to validate the PGRMC1 expression whether influenced by MG-132 or GS-143, the immunoblotting was performed. Compared to the erlotinib treatment, neither the co-treatment of GS-143 with erlotinib (Additional file 1: Fig. S7a) nor MG-132 with erlotinib (Additional file 1: Fig. S7b) showed effect on expression of PGRMC1 inhibition in PC9/ER cells. These results reveal that PGRMC1 in PC9/ER cells activates the NF-κB pathway via the Wnt/β-catenin pathway by enhancing the levels of β-TrCP, leading to erlotinib resistance in these cells.

## Discussion

Here we present evidence to support that PGRMC1 plays an essential role in an acquired erlotinib resistance model, PC9/ER cells. As a new mechanism of EGFR-TKI resistance, we show that PGRMC1 in PC9/ER cells attenuated the efficiency of erlotinib by binding with EGFR, which in turn upregulated the NF-κB pathway via the Wnt/β-catenin pathway through modulation of β-TrCP levels. The expression of PGRMC1 in cells lines resistant to another EGFR-TKI, osimertinib, was higher than in its sensitive cell line, which showed similar patterns to that in the erlotinib-sensitive and -resistant cells lines (Additional file 1: Fig. S8). Further experiments with PGRMC1 inhibitors in osimertinib-sensitive and -resistant cell lines are required.

Inhibition of PGRMC1 by siRNA or AG-205 in PC9/ER cells significantly increased their sensitivity to erlotinib; the effect was not such dramatic for the siRNA (Fig. 1c). PGRMC1 was reported to play many functions for cell stability, including regulation of cytochrome P450, steroidogenesis, vesicle trafficking, progesterone signaling, and mitotic spindle and cell cycle regulation. We revealed that siPGRMC1 showed the significantly decrease compared to cells without erlotinib nor siRNA in both cell lines, which indicated that PGRMC1 plays functions in cell regulation (data not shown). Therefore, to exclude the effect from PGRMC1 fundamental function in cells, the cell viability was compared with each corresponding non-treat group, rather than set the same non-treat group for every group and led to the less difference between groups.

On the other hand, while the results of immunoblotting showed efficient transfection with pCMV3-PGRMC1, only a subtle but significant increase in viability was achieved when PC9 cells were made to overexpress PGRMC1 by transfection with this plasmid (Fig. 1e). Because AG-205 alters the spectroscopic properties of the PGRMC1-heme complex^19^, it is reasonable to infer that the heme participates in key processes, and the heme-PGRMC1 complex may be essential in the mechanism of acquired resistance to erlotinib. However, no difference effect on viability was observed in PC9 and PC9/ER cells when they were treated with succinylacetone plus erlotinib rather than erlotinib alone (Additional file 1: Fig. S3), indicating that unlike PGRMC1-heme complex inhibition, inhibition of heme biosynthesis was not the direct way to overcome erlotinib resistance. By binding to a site in the heme residue of PGRMC1, NO or CO can interfere with the PGRMC1 dimerization required for interaction with EGFR^17^; we therefore consider it likely that the binding ability between PGRMC1 and EGFR is responsible for erlotinib resistance. Our results lead us to hypothesize that PGRMC1 and erlotinib compete for the same binding position with EGFR. Thus, further precisely analyze is demanded to test this hypothesis for completely clarification of erlotinib resistance.

Our results revealed that the NF-κB pathway might contribute to acquired resistance to erlotinib. Stimulation of this pathway by inhibition of oncogenic mutants of *EGFR* mediates tumor cell survival in cells lines of lung cancer^23^. The target genes of PGRMC1 in the NF-κB pathway remain to be identified.

The causes of upregulation of PGRMC1 in acquired resistance to erlotinib are still obscure. Progesterone may participate in the enhancement of EGFR interacting with progesterone receptor^27^ and progesterone receptor has been reported as a new prognostic factor and possible target for NSCLC^28,29^. PGRMC1 is reported to act like a putative progesterone receptor and to be associated with progesterone-dependent effects: e.g., mediating progesterone-dependent protective effects against cisplatin in ovary cancer^16^, and doxorubicin in breast cancer^30^. Hence, we supposed that progesterone might increase the expression of PGRMC1 during the development of acquired resistance to erlotinib.

## Conclusions

Here, we showed that PGRMC1 induces binding between EGFR and PGRMC1, followed by activating crosstalk between the Wnt/β-catenin and NF-κB pathways in PC9/ER cells. These findings suggest that PGRMC1 contributes to acquired EGFR-TKI resistance in NSCLC and is a potential target for anti-resistance therapy. Although further research is needed, our data help clarifies the mechanisms underlying acquired EGFR-TKI resistance. New combination treatments that inhibit these mechanisms would improve the quality of life of lung cancer patients.

## Supplementary information

Supplementary information.

## Data Availability

Data sharing is not applicable to this article as no datasets were generated or analyzed during the current study.

## References

[1] Wojtalla A, Arcaro A (2011). Targeting phosphoinositide 3-kinase signalling in lung cancer. Crit. Rev. Oncol. Hematol..

[2] Lee CC, Shiao HY, Wang WC, Hsieh HP (2014). Small-molecule EGFR tyrosine kinase inhibitors for the treatment of cancer. Expert Opin. Investig. Drugs.

[3] Normanno N (2006). Epidermal growth factor receptor (EGFR) signaling in cancer. Gene.

[4] Huang L, Fu L (2015). Mechanisms of resistance to EGFR tyrosine kinase inhibitors. Acta. Pharm. Sin. B.

[5] Ramalingam S, Belani C (2008). Systemic chemotherapy for advanced non-small cell lung cancer: recent advances and future directions. Oncologist.

[6] Kobayashi S (2005). EGFR mutation and resistance of non-small-cell lung cancer to gefitinib. N. Engl. J. Med..

[7] Wu SG, Shih JY (2018). Management of acquired resistance to EGFR TKI-targeted therapy in advanced non-small cell lung cancer. Mol. Cancer.

[8] Takezawa K (2012). HER2 amplification: a potential mechanism of acquired resistance to EGFR inhibition in EGFR-mutant lung cancers that lack the second-site EGFRT790M mutation. Cancer Discov..

[9] Yun CH (2008). The T790M mutation in EGFR kinase causes drug resistance by increasing the affinity for ATP. Proc. Natl. Acad. Sci. USA.

[10] Ahmed IS, Rohe HJ, Twist KE, Craven RJ (2010). Pgrmc1 (progesterone receptor membrane component 1) associates with epidermal growth factor receptor and regulates erlotinib sensitivity. J. Biol. Chem..

[11] Oser MG, Niederst MJ, Sequist LV, Engelman JA (2015). Transformation from non-small-cell lung cancer to small-cell lung cancer: molecular drivers and cells of origin. The Lancet Oncology.

[12] Dorantes-Heredia R, Ruiz-Morales JM, Cano-Garcia F (2016). Histopathological transformation to small-cell lung carcinoma in non-small cell lung carcinoma tumors. Transl. Lung Cancer Res..

[13] Shintani T (2018). Eukaryotic translation initiation factor 3 subunit C is associated with acquired resistance to erlotinib in non-small cell lung cancer. Oncotarget.

[14] Ruan X (2017). Increased expression of progesterone receptor membrane component 1 is associated with aggressive phenotype and poor prognosis in ER-positive and negative breast cancer. Menopause.

[15] Mir SU, Ahmed IS, Arnold S, Craven RJ (2012). Elevated progesterone receptor membrane component 1/sigma-2 receptor levels in lung tumors and plasma from lung cancer patients. Int. J. Cancer.

[16] Peluso JJ, Liu X, Saunders MM, Claffey KP, Phoenix K (2008). Regulation of ovarian cancer cell viability and sensitivity to cisplatin by progesterone receptor membrane component-1. J. Clin. Endocrinol. Metab..

[17] Kabe Y (2016). Haem-dependent dimerization of PGRMC1/Sigma-2 receptor facilitates cancer proliferation and chemoresistance. Nat. Commun..

[18] Lin CC (2015). Identification of protein expression alterations in gefitinib-resistant human lung adenocarcinoma: PCNT and mPR play key roles in the development of gefitinib-associated resistance. Toxicol. Appl. Pharmacol..

[19] Ahmed IS, Rohe HJ, Twist KE, Mattingly MN, Craven RJ (2010). Progesterone receptor membrane component 1 (Pgrmc1): a heme-1 domain protein that promotes tumorigenesis and is inhibited by a small molecule. J. Pharmacol. Exp. Ther..

[20] Kim JY (2018). Progesterone Receptor Membrane Component 1 suppresses the p53 and Wnt/beta-catenin pathways to promote human pluripotent stem cell self-renewal. Sci. Rep..

[21] Mir SU, Jin L, Craven RJ (2012). Neutrophil gelatinase-associated lipocalin (NGAL) expression is dependent on the tumor-associated sigma-2 receptor S2RPgrmc1. J. Biol. Chem..

[22] Nakata A (2015). Elevated beta-catenin pathway as a novel target for patients with resistance to EGF receptor targeting drugs. Sci. Rep..

[23] Blakely CM (2015). NF-kappaB-activating complex engaged in response to EGFR oncogene inhibition drives tumor cell survival and residual disease in lung cancer. Cell Rep..

[24] Schwitalla S (2013). Intestinal tumorigenesis initiated by dedifferentiation and acquisition of stem-cell-like properties. Cell.

[25] Meyer L (2007). beta-Trcp mediates ubiquitination and degradation of the erythropoietin receptor and controls cell proliferation. Blood.

[26] Ma, B. & Hottiger, M. O. Crosstalk between Wnt/β-Catenin and NF-κB Signaling Pathway during Inflammation. *Front. Immunol*. **7** (2016). 10.3389/fimmu.2016.00378 PMC5031610 27713747

[27] Tania Hernandez-Hernandez O, Camacho-Arroyo I (2013). Regulation of Gene Expression by Progesterone in Cancer Cells: Effects on Cyclin D1, EGFR and VEGF. Mini-Rev. Med. Chem..

[28] Ishibashi H (2005). Progesterone receptor in non-small cell lung cancer–a potent prognostic factor and possible target for endocrine therapy. Cancer Res..

[29] Marquez-Garban DC (2011). Progesterone and estrogen receptor expression and activity in human non-small cell lung cancer. Steroids.

[30] Crudden G, Chitti RE, Craven RJ (2006). Hpr6 (heme-1 domain protein) regulates the susceptibility of cancer cells to chemotherapeutic drugs. J. Pharmacol. Exp. Ther..

