# Supplementary material for: Progesterone receptor membrane component 1 leads to erlotinib resistance, initiating crosstalk of Wnt/β-catenin and NF-κB pathways, in lung adenocarcinoma cells
Source: Sci Rep. 2020 Mar 16;10:4748. doi: 10.1038/s41598-020-61727-3 (PMC7076038; doi:10.1038/s41598-020-61727-3)

Title

Progesterone receptor membrane component 1 leads to erlotinib resistance, initiating crosstalk of Wnt/β-catenin and NF-κB pathways, in lung adenocarcinoma cells

Authors

Ying Lin[a,1], Kazuma Higashisaka[a,b,1,†], Takuya Shintani[c,d], Ayaka Maki[a], Sachiyo Hanamuro[a], Yuya Haga[a], Shinichiro Maeda[c,d], Hirofumi Tsujino[a], Kazuya Nagano[a], Yasushi Fujio[d,e], Yasuo Tsutsumi[a,f,†]

Affiliations

[a]Laboratory of Toxicology and Safety Science, Graduate School of Pharmaceutical Sciences, Osaka University, 1-6 Yamadaoka, Suita, Osaka 565-0871, Japan.

[b]Department of Legal Medicine, Osaka University Graduate School of Medicine, 2-2 Yamadaoka, Suita, Osaka 565-0871, Japan.

[c]Department of Pharmacy, Osaka University Hospital, 2-15 Yamadaoka, Suita, Osaka 565-0871, Japan.

[d]Advanced Research of Medical and Pharmaceutical Sciences, Graduate School of Pharmaceutical Sciences, Osaka University, 1-6 Yamadaoka, Suita, Osaka 565-0871, Japan.

[e]Laboratory of Clinical Science and Biomedicine, Graduate School of Pharmaceutical Sciences, Osaka University, 1-6 Yamadaoka, Suita, Osaka 565-0871, Japan

[f]Global Center for Medical Engineering and Informatics, Osaka University, 2-2 Yamadaoka, Suita, Osaka 565-0871, Japan.

[1]These authors contributed equally to the work.

E-mail addresses

Ying Lin: lin-y@phs.osaka-u.ac.jp; Kazuma Higashisaka: higashisaka@legal.med.osaka-u.ac.jp; Takuya Shintani: shintani@hosp.med.osaka-u.ac.jp; Ayaka Maki: ayaka.mk56@gmail.com; Sachiyo Hanamuro: hanamuro-s@phs.osaka-u.ac.jp; Yuya Haga: haga-y@phs.osaka-u.ac.jp; Shinichiro Maeda: maeda313@phs.osaka-u.ac.jp; Hirofumi Tsujino: htsujino@phs.osaka-u.ac.jp; Kazuya Nagano: knagano@phs.osaka-u.ac.jp; Yasushi Fujio: fujio@phs.osaka-u.ac.jp; Yasuo Tsutsumi: ytsutsumi@phs.osaka-u.ac.jp

Corresponding Authors

[†]To whom correspondence should be addressed.

Kazuma Higashisaka, PhD

Department of Legal Medicine, Osaka University Graduate School of Medicine
2-2, Yamadaoka, Suita, Osaka 565-0871, Japan
Tel: +81-6-6879-3112
Fax: +81-6-6879-3119
E-mail address: higashisaka@legal.med.osaka-u.ac.jp

Yasuo Tsutsumi, PhD

Laboratory of Toxicology and Safety Science, Graduate School of Pharmaceutical Sciences, Osaka University
1-6, Yamadaoka, Suita, Osaka 565-0871, Japan
Tel: +81-6-6879-8230
Fax: +81-6-6879-8234
E-mail address: ytsutsumi@phs.osaka-u.ac.jp

MATERIALS AND METHODS

Clinical analysis

Kaplan Meier Plotter was established using PGRMC1 gene expression data and survival information of lung cancer patients downloaded from Gene Expression Omnibus. PGRMC1 gene (probe set, 201120_at) was entered into the database (http://kmplot.com/lung, last accessing time 2020/01/20) to obtain the Kaplan-Meier survival plot. Genome alternation of PGRMC1 in NSCLC was obtaind from OncoPrint outputs (cBioPortal for Cancer Genomics; www.cbioportal.org, last accessing time 2019/11/28).

Cell lines and cell cultures

The NSCLC cell line NCI-H1975 (osimertinib-sensitive) and NCI-H1975/OR (osimertinib-resistant) were kindly gifted by Dr. Kinehara (Osaka University, Japan). NCI-H1975 cells were cultured in RPMI-1640 (Wako, Osaka, Japan) with 10% (v/v) fetal bovine serum (Gibco, Waltham, MA, USA) and 1% (v/v) Antibiotic-Antimycotic (100X) (Thermo Fisher Scientific, Waltham, MA, USA) and maintained at 37° C with 5% CO2 and over 95% humidity. NCI-H1975/OR cells were cultured in the same conditions as the NCI-H1975 cells with the addition of 800 nM osimertinib (Selleckchem, Houston, TX, USA).

Cell Viability

PC9 and PC9/ER cells were seeded at 8000 cells/well without drugs in 96-well flat plates overnight; the medium was then replaced and the cells were treated with 20 nM erlotinib (Selleckchem) or 0.1, 1, or 10 mM succinylacetone (Sigma-Aldrich) for 72 h. Cell viability was evaluated by WST-8 assay (Nacalai Tesque, Inc., Kyoto, Japan). Erlotinib and succinylacetone were dissolved in DMSO and stored at −20°C prior to use.

Transfection of PGRMC1 vector

pCMV3 plasmid encoding human PGRMC1 was purchased from Sino Biological Inc. (Beijing, China). The insert cDNA contained the complete PGRMC1 coding sequence (NM_006667.4). The pCMV3-PGRMC1 and pCMV3 negative control vectors were separately transfected into PC9 cells at 70%–80% confluency using Lipofectamine 2000 Transfection Reagent (Invitrogen) for 24 h in RPMI-1640 containing 10% FBS.

Transient transfection of Negative Control and/or PGRMC1 small interfering RNA (NC and/or PGRMC1 siRNA)

Cells were transfected with 50 nM Stealth siRNA against PGRMC1 (5′-GGGAGUCUCAGUUCAUUUtt-3′ and 3′AAAGUGAACUGACUCCCag-5′) or Stealth siRNA negative control with medium GC content (Invitrogen, Carlsbad, CA, USA). The transfection was executed with Lipofectamine RNAiMAX Transfection Reagent (Invitrogen) for 24 h in RPMI-1640 containing 10% FBS and 1% Ab.

MATERIALS AND METHODS (Continue)

Immunoblotting analysis

Proteins were extracted by using M-PER Mammalian Protein Extraction Reagent (Thermo Fisher Scientific) with Protease Inhibitor Cocktail (Thermo Fisher Scientific). Nucleus proteins were extracted by using NE-PER™ Nuclear and Cytoplasmic Extraction Reagents (Thermo Fisher Scientific) with Protease Inhibitor Cocktail.Proteins were mixed with an equal volume of Laemmli sample buffer (Bio-Rad Laboratories, Hercules, CA, USA) containing 5% 2-mercaptoethanol and then boiled for 5 min prior to separation by sodium dodecyl sulfate polyacrylamide gel electrophoresis. Precision Plus Protein Kaleidoscope molecular weight markers (Bio-Rad laboratories) were used as standards. The proteins were then electro-transferred onto a polyvinylidene difluoride membrane (Millipore, Bedford, MA) and blocked with 4% Block Ace (KAC Co., Ltd., Kyoto, Japan) in phosphate-buffered saline plus 0.01% (v/v) Triton X-100 (PBST). The membranes were incubated with primary antibody for 1 h and then treated with secondary antibody for 1 h. The following antibodies were used: anti-PGRMC1 monoclonal antibody (mAb) (#13856, 1:1000), anti-epidermal growth factor receptor (EGFR) mAb (#4267, 1:1000), anti-phospho-EGFRTyr1068 mAb (#3777, 1:1000), anti-phospho-EGFRTyr992 mAb (#2235, 1:500), anti-phospho-EGFRTyr845 mAb (#6963, 1:500), anti-Akt mAb (#4691, 1:2000), anti-phospho-AktSer473 mAb (#4060, 1:2000), anti-ERK pAb (#9102, 1:2000), anti-phospho-ERKT202/Y204 pAb(#9101, 1:2000), anti-Lamin A/C pAb (#2032, 1:2000)and anti-rabbit IgG-horseradish peroxidase-conjugated secondary antibody (#7074S, 1:2000) purchased from Cell Signaling Technology (Danvers, MA, USA); and anti-β-actin mAb (A5316,1:50000) and anti-mouse IgG secondary antibody purchased from Sigma-Aldrich (Darmstadt, DE, USA), anti-β-catenin mAb (sc-7963, 1:1000) was purchase from Santa Cruz Biotechnology, Inc., (Dallas, TX, USA). All of them were diluted in 0.4% Block Ace in PBST. The protein bands were detected by using SuperSignalWest Femto Maximum Sensitivity Substrate (Thermo Fisher Scientific) and visualized with an ImageQuant LAS 4000 mini biomolecular imager (GE Healthcare Japan, Tokyo, Japan). The resultant images were analyzed by using ImageJ JAVA 1.6.0_24 (64-bit) (National Institutes of Health, Bethesda, MD, USA).

(a)

(b)

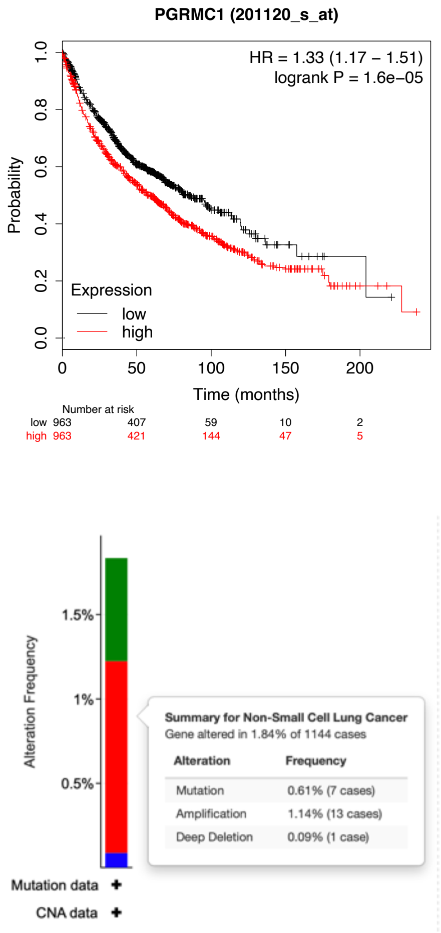

Figure S1. Clinical relevance of PGRMC1 in lung cancer. (a) Kaplan-Meier plots derived from http://kmplot.com/analysis/. The analyses were performed and the Kaplan-Meier plots were drawn regarding PGRMC1 expression and overall survival in the lung cancer. (b) Genome alternation of PGRMC1 in NSCLC subtypes. Shown are OncoPrint outputs (cBioPortal for Cancer Genomics database; www.cbioportal.org) where each bar represents a tumor that was found to contain a DNA alteration (13 cases amplification as indicated in red, 1 case deletion as indicated in blue, 7 cases mutation, as indicated in green) in PGRMC1.

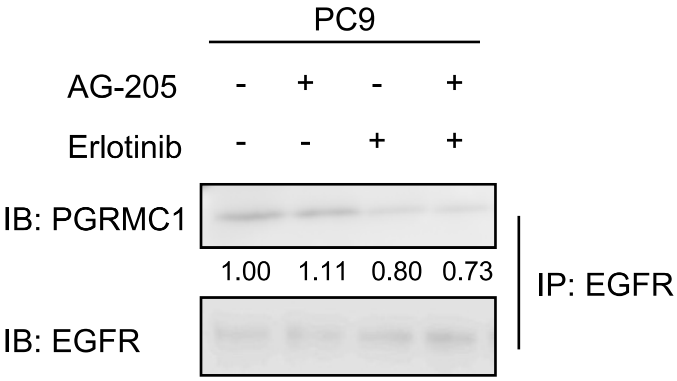

Figure S2. Effects of combination treatment of erlotinib and AG-205 on EGFR–PGRMC1 binding in lung adenocarcinoma cells. Immunoprecipitation assay of binding between EGFR and PGRMC1 in PC9 cells cotreated with AG-205 (1 μM) plus erlotinib (20 nM) for 72 h.

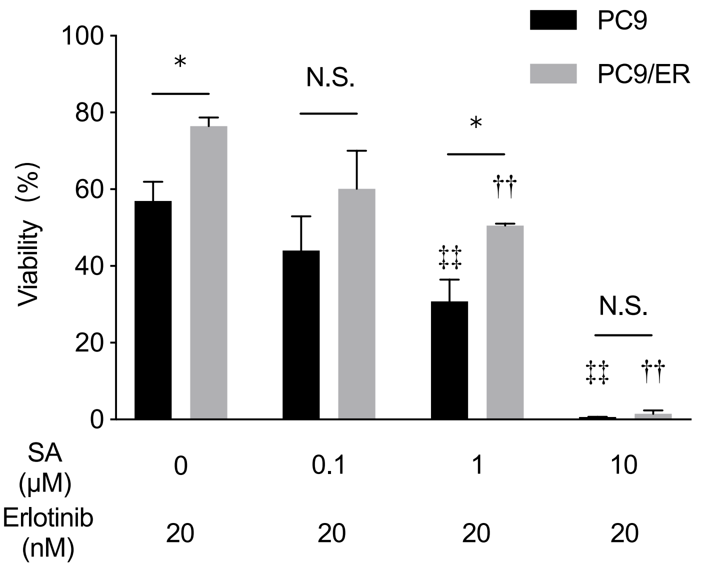

Figure S3. Effects of combination treatment of erlotinib and succinylacetone on cell viability. PC9 and PC9/ER cells were cotreated with succinylacetone (SA) plus erlotinib for 72 h, and then cell viability was monitored by WST-8 assay. Data are means ± SD (n = 3); **P* < 0.05 PC9 vs. PC9/ER, ††*P* < 0.01 vs. PC9/ER cells treated with erlotinib alone, ‡‡*P* < 0.01 vs. PC9 cells treated with erlotinib alone (Two-Way ANOVA followed by Bonferroni correction), N.S; not significant.

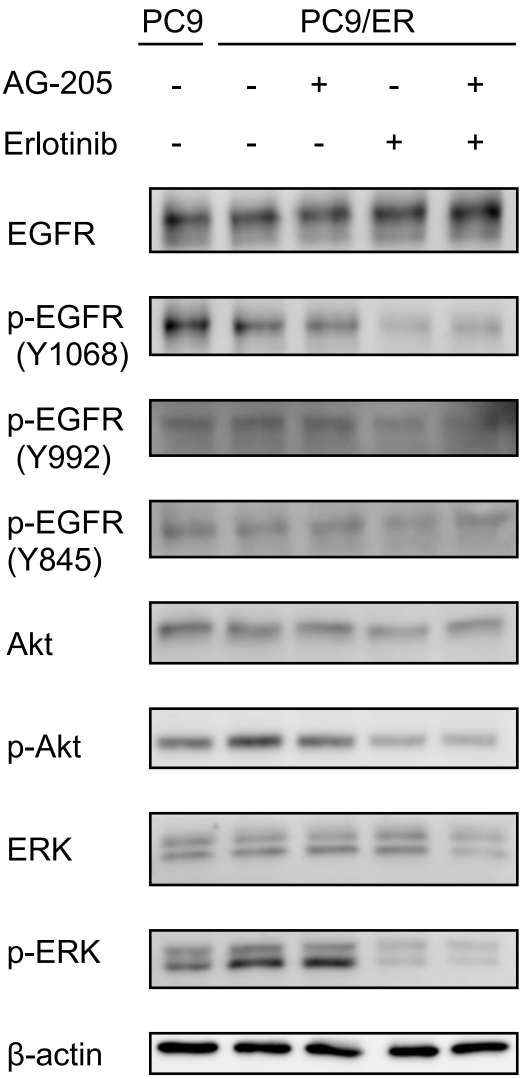

Figure S4. Effects of combination treatment of erlotinib and AG-205 on EGFR in lung adenocarcinoma cells. Activation of EGFR (Y1068, Y992, and Y845), Akt, and ERK by combination treatment of AG-205 plus erlotinib were assessed by immunoblotting.

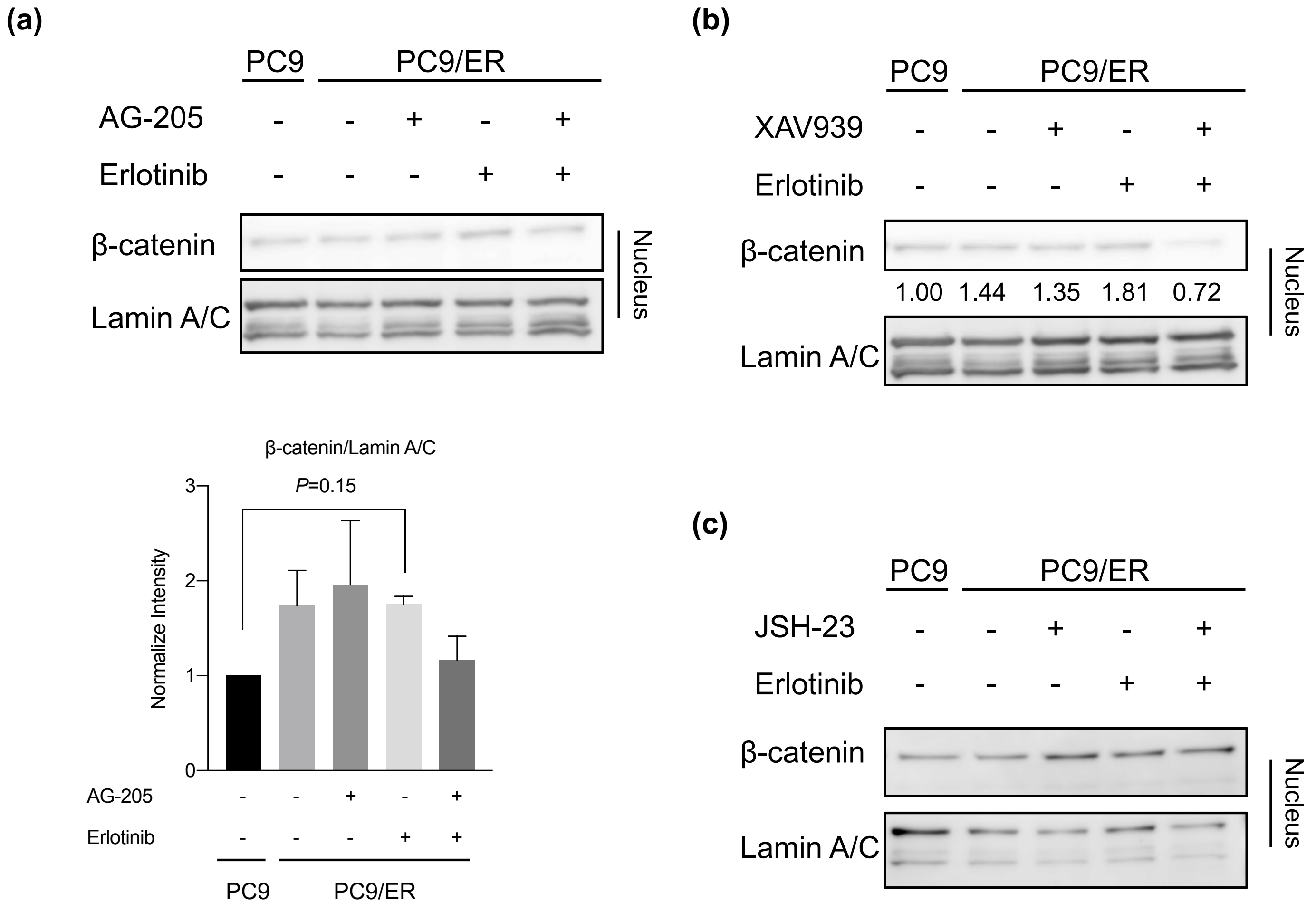

Figure S5. Effects of combination treatment of erlotinib and AG-205 or XAV939 or JSH-23 on nuclear β-catenin in lung adenocarcinoma cells. (a) Nucleus β-catenin by combination treatment of AG-205 (1 μM) plus erlotinib (20 nM) were assessed by immunoblotting. Data are means ± SD (n = 3); One-Way ANOVA followed by Tukey correction. (b) Nucleus β-catenin by combination treatment of XAV939 (10 μM) plus erlotinib (20 nM) were assessed by immunoblotting. (c) Nucleus β-catenin by combination treatment of JSH-23 (10 μM) plus erlotinib (20 nM) were assessed by immunoblotting.

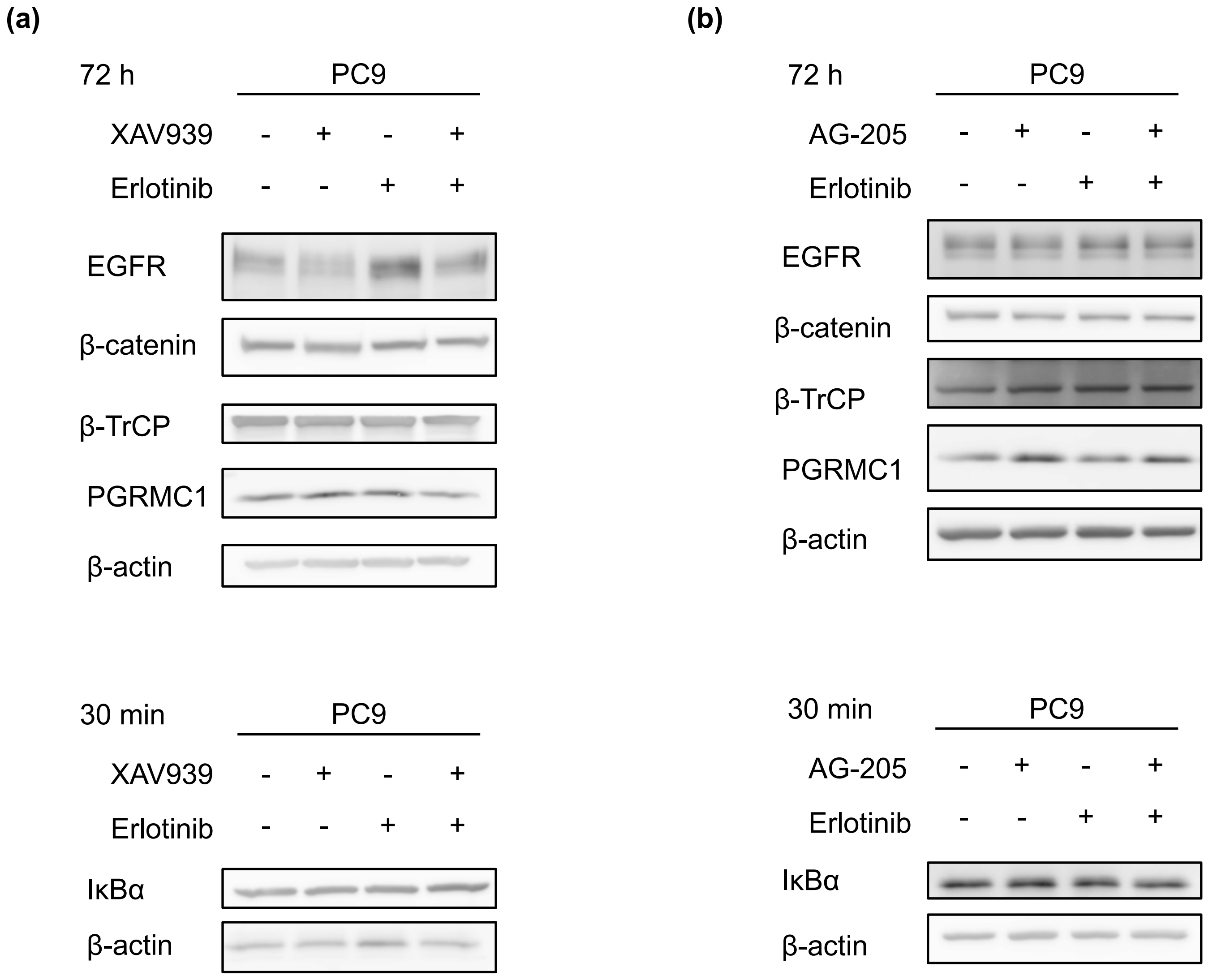

Figure S6. Effects of combination treatment of erlotinib and AG-205 or XAV939 on protein expression in PC9 cells. (a) EGFR, β-catenin, β-TrCP, PGRMC1, and IκBα (30 min) by combination treatment of XAV939 (10 μM) plus erlotinib (20 nM) were assessed by immunoblotting. (b) EGFR, β-catenin, β-TrCP, PGRMC1, and IκBα (30 min) in whole lysate by combination treatment of AG-205 (1 μM) plus erlotinib (20 nM) were assessed by immunoblotting.

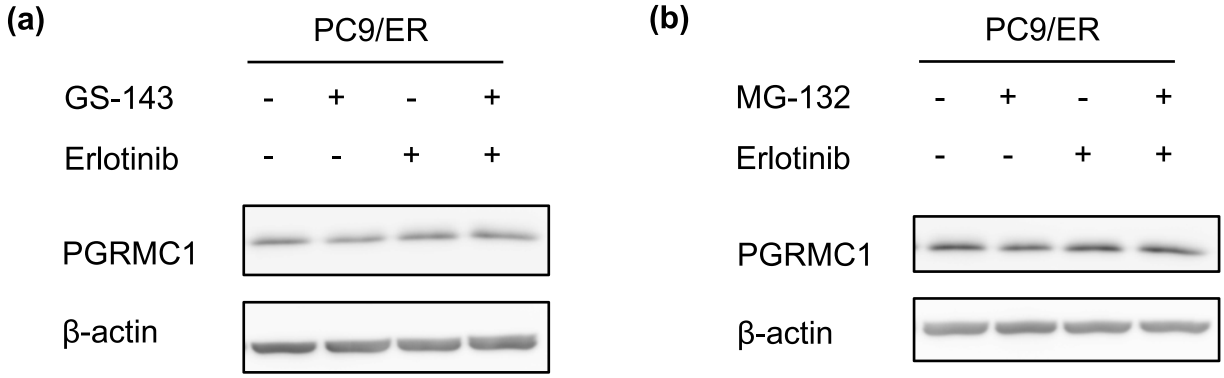

Figure S7. Effects of combination treatment of erlotinib and GS-143 or MG-132 on PGRMC1 in lung adenocarcinoma cells. (a) PGRMC1 by combination treatment of GS-143 (2 μM) plus erlotinib (20 nM) were assessed by immunoblotting. (b) PGRMC1 by combination treatment of MG-132 (0.01 μM) plus erlotinib (20 nM) were assessed by immunoblotting.

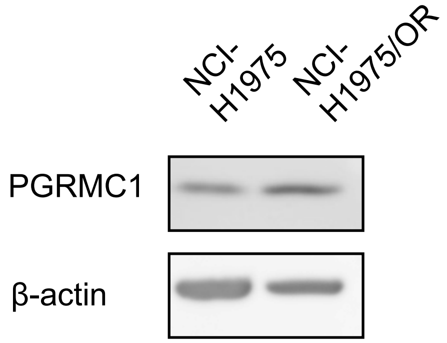

Figure S8. Comparison of PGRMC1 expression in osimertinib-sensitive (NCI-H1975) and osimertinib-resistant (NCI-H1975/OR) lung adenocarcinoma cancer cells. PGRMC1 expression was monitored by immunoblotting.

1. Original western blots cropped in Figure 1

All western blots were cropped using Microsoft PowerPoint. We did not use any software to increase the contrast. The low background is a result of using 4% Block Ace for blocking solution, first, and secondary antibody. All bands from one figure origin from the same gel/membrane that was horizontally cut and exposed to different primary antibodies.

1.1. PGRMC1 and β-actin

Blot used in figure 1a. We detected PGRMC1 and β-actin at the same membrane using the different primary antibody.

PGRMC1

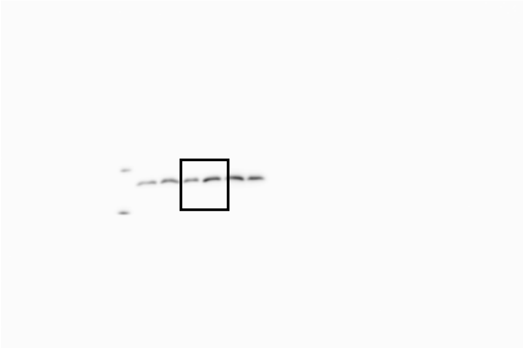

β-actin

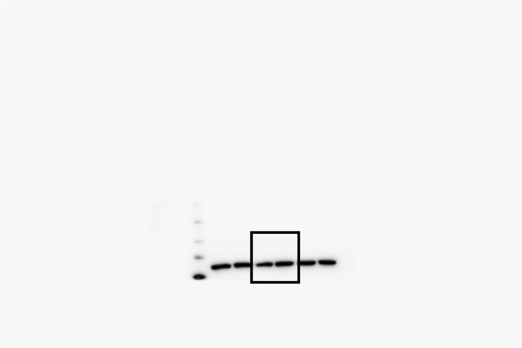

1.2. PGRMC1 and β-actin

Blot used in figure 1b. We detected PGRMC1 and β-actin at the same membrane using the different primary antibody.

PGRMC1

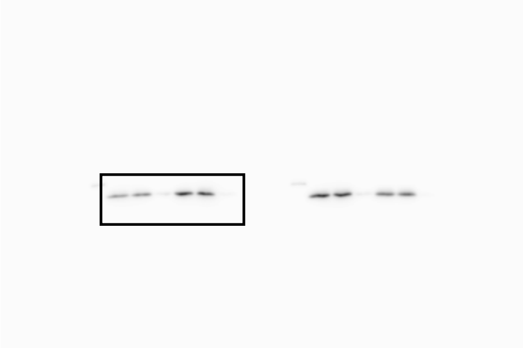

β-actin

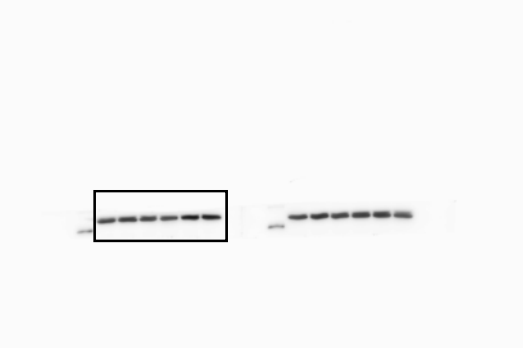

1.3. PGRMC1 and β-actin

Blot used in figure 1d. We detected PGRMC1 and β-actin at the same membrane using the different primary antibody.

PGRMC1

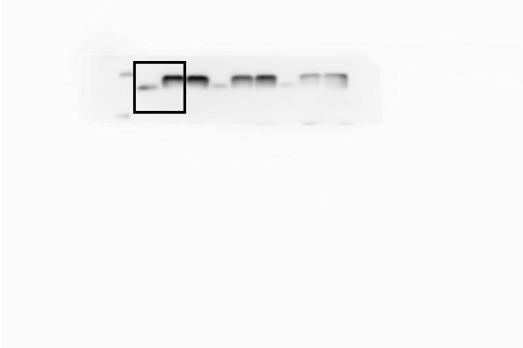

β-actin

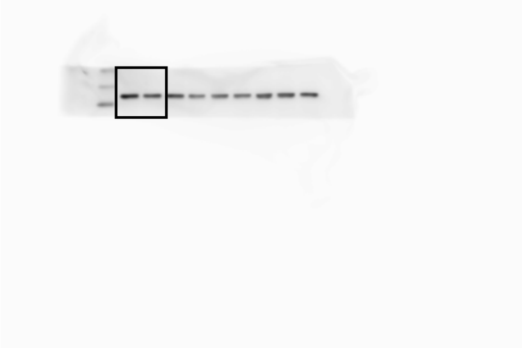

2. Original western blots cropped in Figure 2

All western blots were cropped using Microsoft PowerPoint. We did not use any software to increase the contrast. The low background is a result of using 4% Block Ace for blocking solution, first, and secondary antibody. All bands from one figure origin from the same gel/membrane that was horizontally cut and exposed to different primary antibodies.

2.1. EGFR and PGRMC1

Blot used in figure 2a. We detected EGFR and PGRMC1 at the same membrane using the different primary antibody.

EGFR

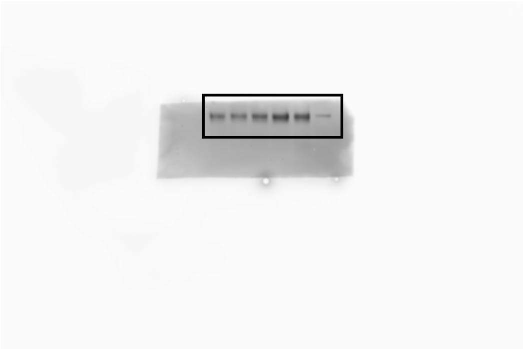

PGRMC1

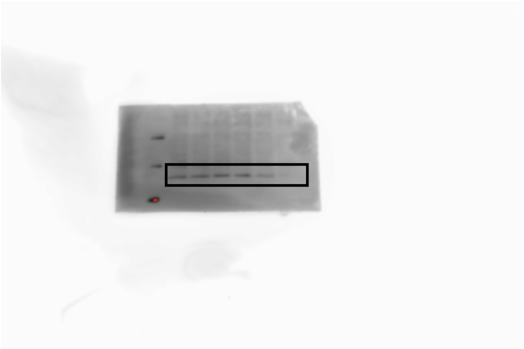

EGFR

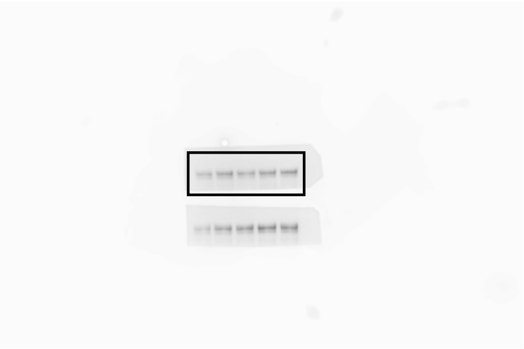

PGRMC1

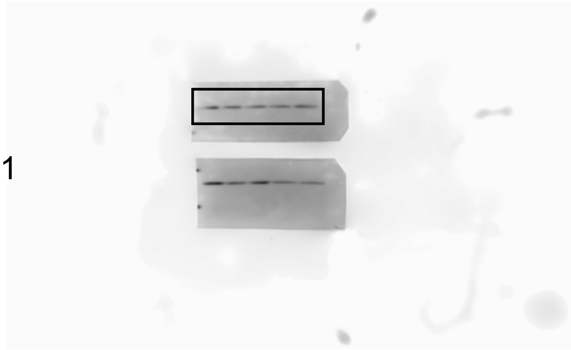

β-actin

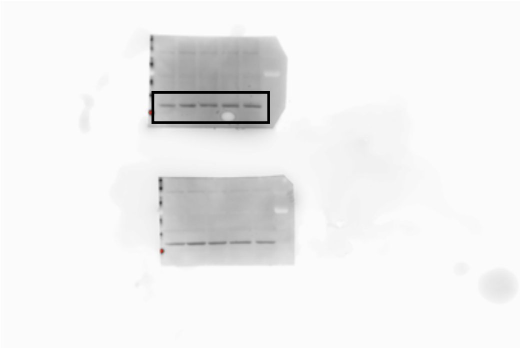

3. Original western blots cropped in Figure 3

All western blots were cropped using Microsoft PowerPoint. We did not use any software to increase the contrast. The low background is a result of using 4% Block Ace for blocking solution, first, and secondary antibody. All bands from one figure origin from the same gel/membrane or different gel/membrane with same sample that was horizontally cut and exposed to different primary antibodies.

3.1. β-catenin and β-actin

Blot used in figure 3a. We detected β-catenin and β-actin at the same membrane using the different primary antibody.

β-catenin

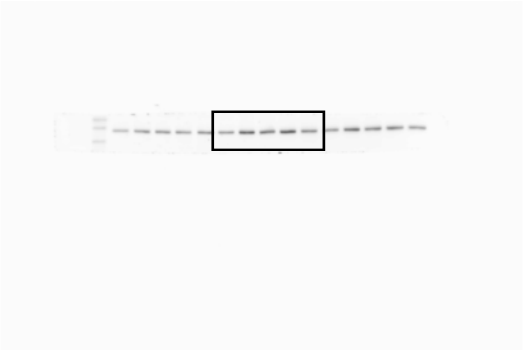

β-actin

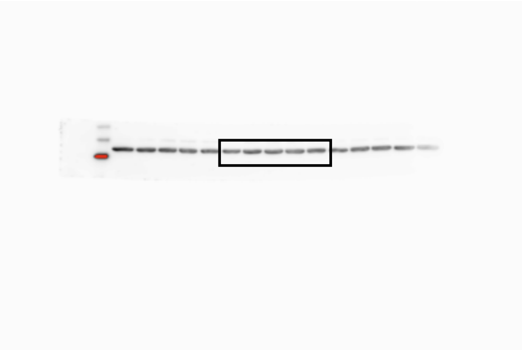

3.2. β-catenin, PGRMC1 and β-actin

Blot used in figure 3b. We detected β-catenin, PGRMC1 and β-actin with the same sample set at the different membrane using the different primary antibody.

β-catenin

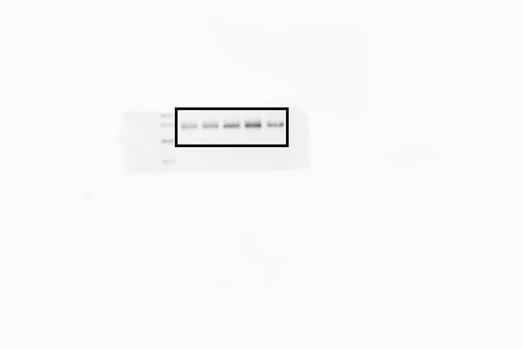

β-actin

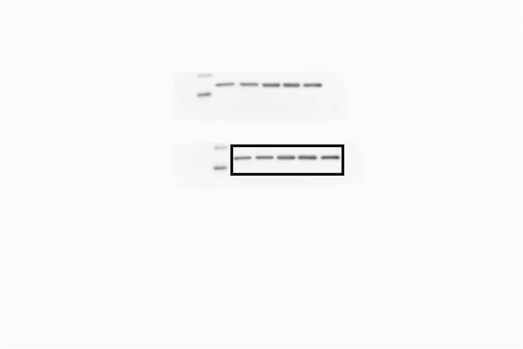

PGRMC1

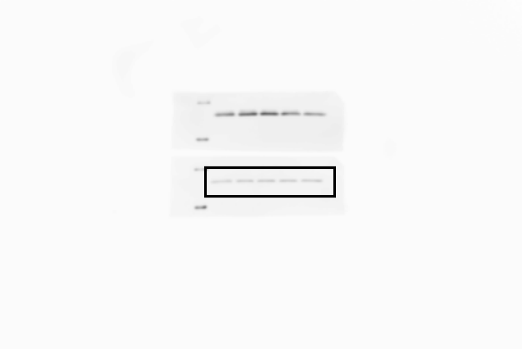

β-actin

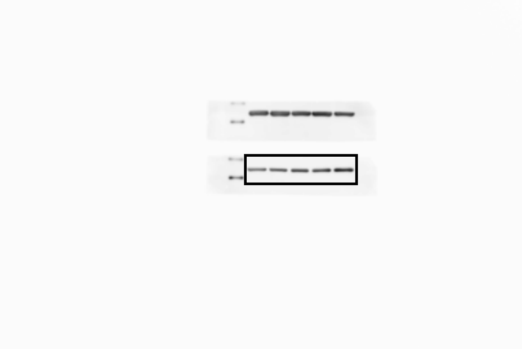

3.3. IκBα, PGRMC1 and β-actin

Blot used in figure 3d. We detected IκBα, PGRMC1 and β-actin at the different membrane with the same sample set using the different primary antibody.

IκBα

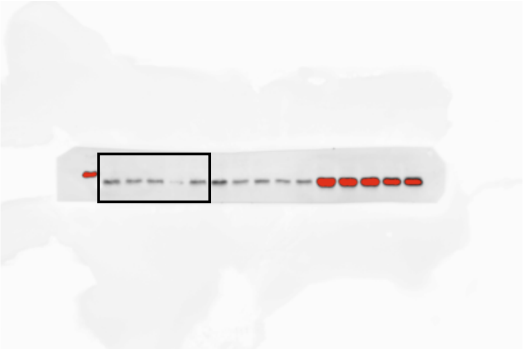

β-actin

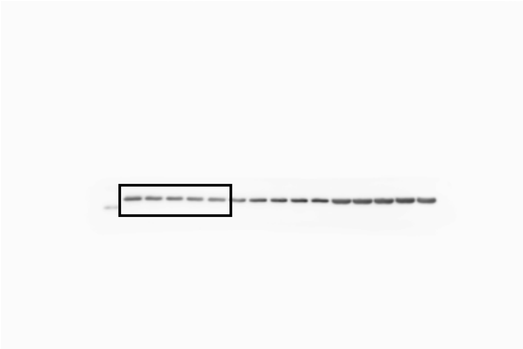

PGRMC1

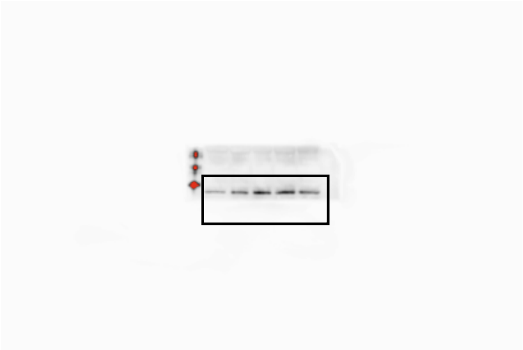

β-actin

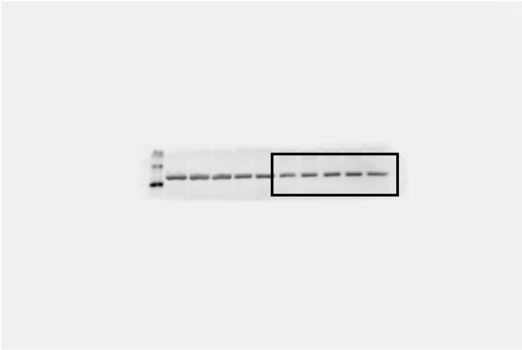

4. Original western blots cropped in Figure 4

All western blots were cropped using Microsoft PowerPoint. We did not use any software to increase the contrast. The low background is a result of using 4% Block Ace for blocking solution, first, and secondary antibody All bands from one figure origin from the same gel/membrane or different gel/membrane with same sample that was horizontally cut and exposed to different primary antibodies.

4.1. β-TrCP and β-actin

Blot used in figure 4a. We detected β-TrCP and β-actin at the same membrane using the different primary antibody.

β-TrCP

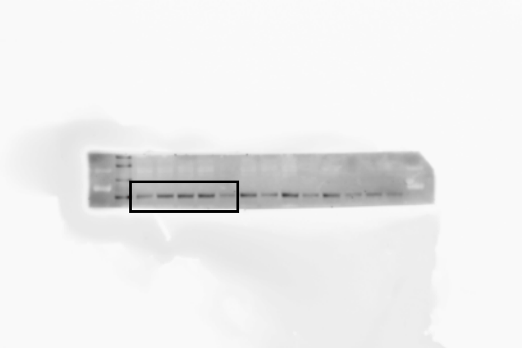

β-actin

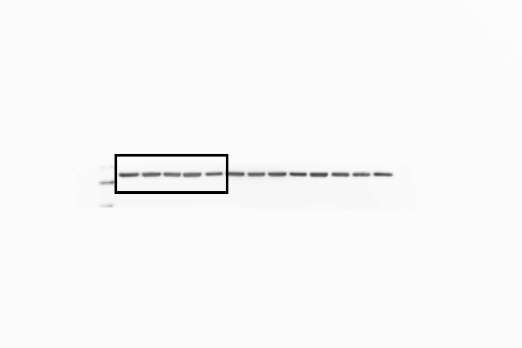

4.2. β-TrCP and β-actin

Blot used in figure 4b. We detected β-TrCP and β-actin at the same membrane using the different primary antibody.

β-TrCP

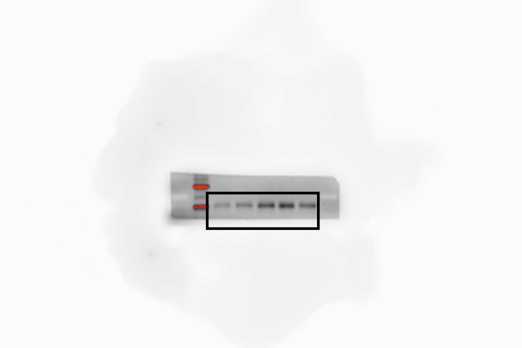

β-actin

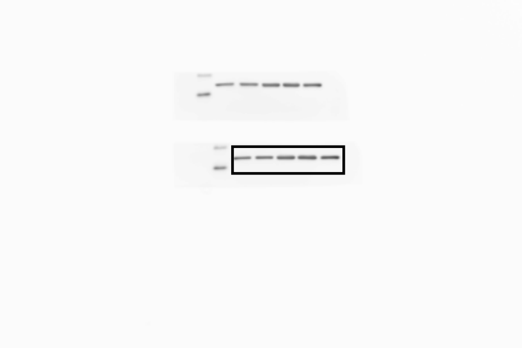

4. Original western blots cropped in Figure 4 (Continue)

All western blots were cropped using Microsoft PowerPoint. We did not use any software to increase the contrast. The low background is a result of using 4% Block Ace for blocking solution, first, and secondary antibody All bands from one figure origin from the same gel/membrane or different gel/membrane with same sample that was horizontally cut and exposed to different primary antibodies.

4.3. IκBα and β-actin

Blot used in figure 4b. We detected IκBα and β-actin at the different membrane with the same sample set using the different primary antibody.

IκBα

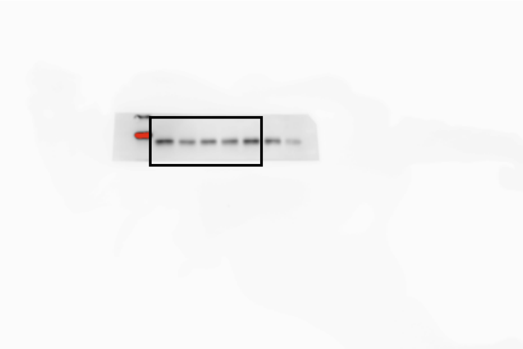

β-actin

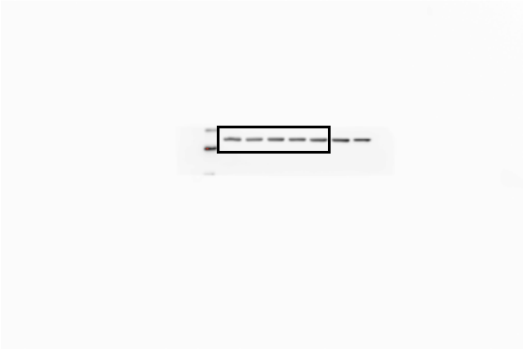

4.4. β-catenin and β-actin

Blot used in figure 4c. We detected β-catenin and β-actin at the same membrane using the different primary antibody.

β-catenin

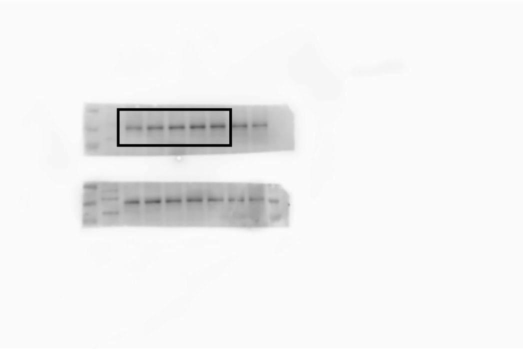

β-actin

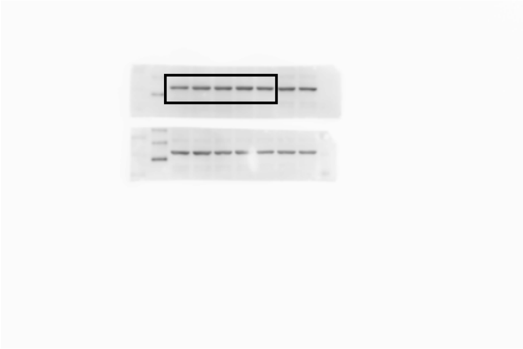

5. Original western blots cropped in Supplementary Figure 2

All western blots were cropped using Microsoft PowerPoint. We did not use any software to increase the contrast. The low background is a result of using 4% Block Ace for blocking solution, first, and secondary antibody. All bands from one figure origin from the same gel/membrane that was horizontally cut and exposed to different primary antibodies.

5.1 PGRMC1 EGFR

Blot used in supplementary Figure 2. We detected PGRMC1 and EGFR at the same membrane using the different primary antibody.

EGFR

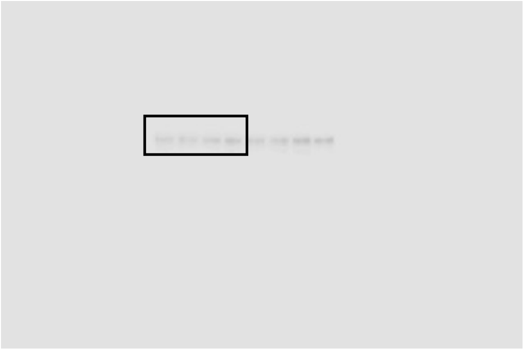

PGRMC1

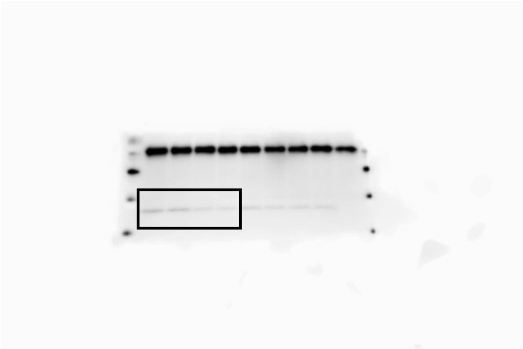

6. Original western blots cropped in Supplementary Figure 4

All western blots were cropped using Microsoft PowerPoint. We did not use any software to increase the contrast. The low background is a result of using 4% Block Ace for blocking solution, first, and secondary antibody. All bands from one figure origin from the same gel/membrane or different gel/membrane with same sample that was horizontally cut and exposed to different primary antibodies.

6.1. EGFR, p-EGFR (Y1068), p-EGFR (Y845), p-EGFR (Y992), Akt, p-Akt, ERK, p-ERK, β-actin

Blot used in supplementary Figure 4. We detected EGFR, p-EGFR (Y1068), p-EGFR (Y845), p-EGFR (Y992), Akt, p-Akt, ERK, p-ERK, and β-actin at the different membrane with the same sample set using the different primary antibody.

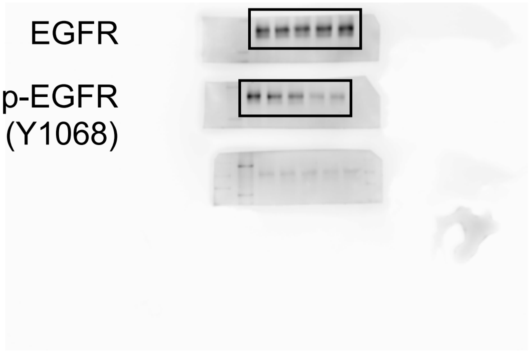

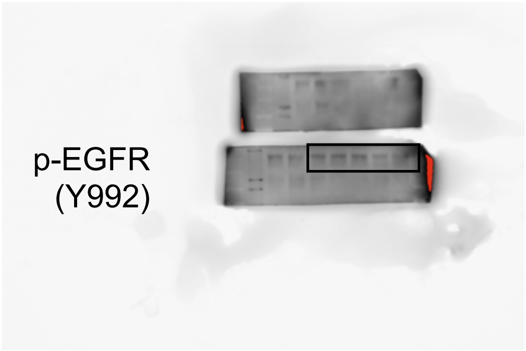

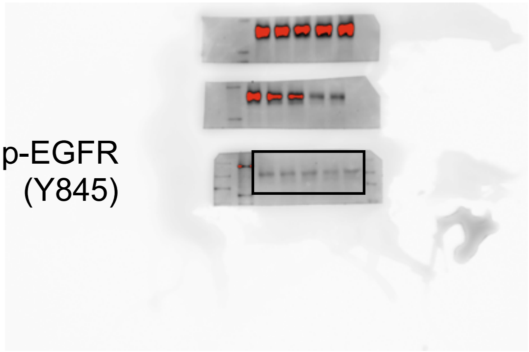

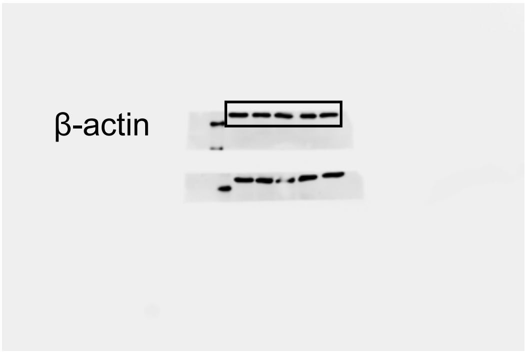

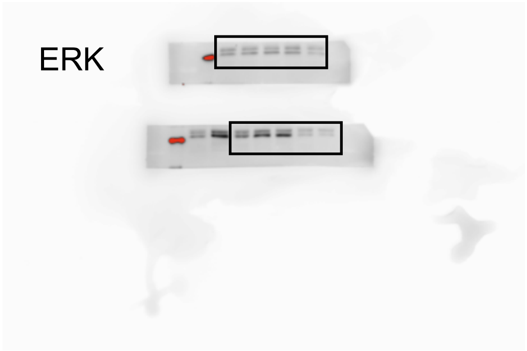

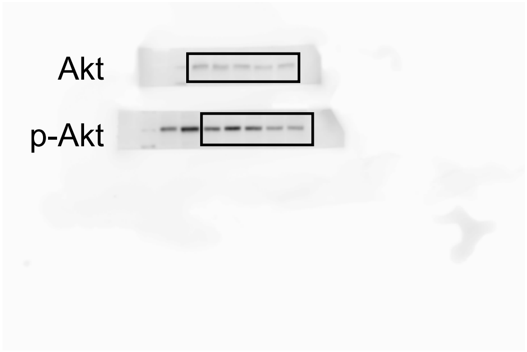

7. Original western blots cropped in Supplementary Figure 5

All western blots were cropped using Microsoft PowerPoint. We did not use any software to increase the contrast. The low background is a result of using 4% Block Ace for blocking solution, first, and secondary antibody. All bands from one figure origin from the same gel/membrane or different gel/membrane with same sample that was horizontally cut and exposed to different primary antibodies.

7.1. β-catenin and Lamin A/C

Blot used in Supplementary Figure 5a. We detected β-catenin and Lamin A/C at the same membrane using the different primary antibody.

β-catenin

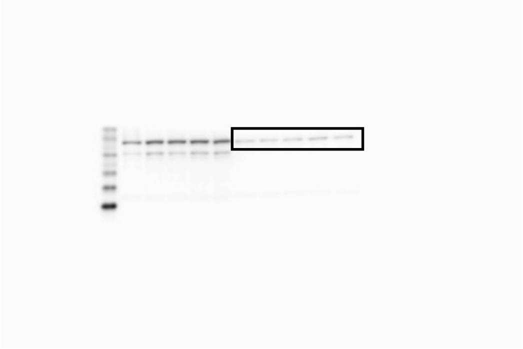

Lamin A/C

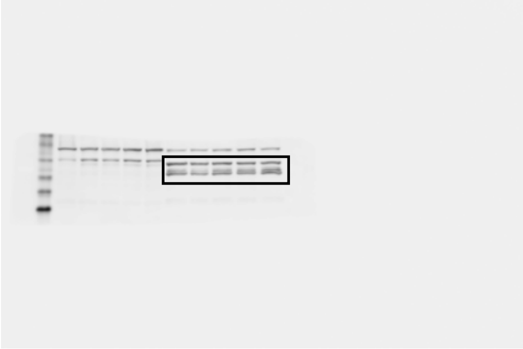

3.2. β-catenin and Lamin A/C

Blot used in Supplementary Figure 5b. We detected β-catenin and Lamin A/C at the same membrane using the different primary antibody.

β-catenin

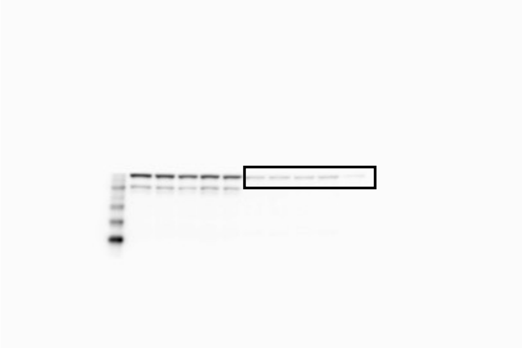

Lamin A/C

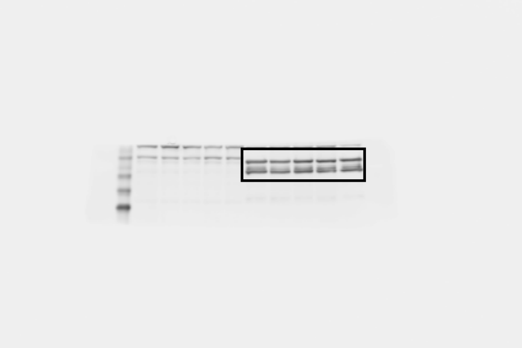

3.3. β-catenin and Lamin A/C

Blot used in Supplementary Figure 5c. We detected β-catenin and Lamin A/C at the same membrane using the different primary antibody.

β-catenin

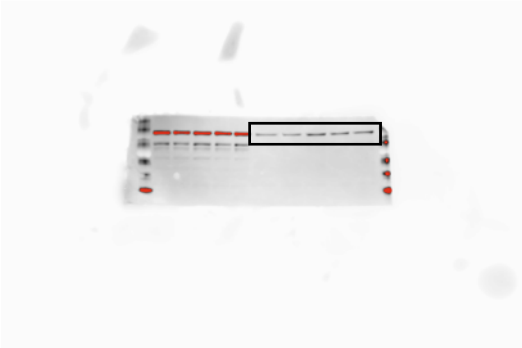

Lamin A/C

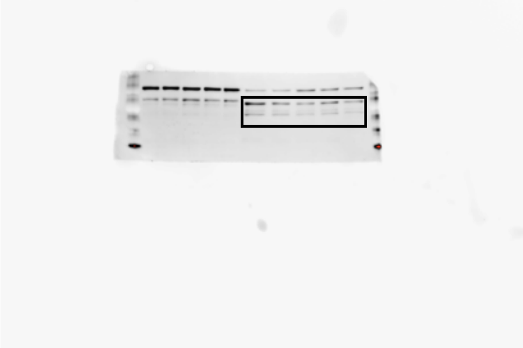

8. Original western blots cropped in Supplementary Figure 6
All western blots were cropped using Microsoft PowerPoint. We did not use any software to increase the contrast. The low background is a result of using 4% Block Ace for blocking solution, first, and secondary antibody. All bands from one figure origin from the same gel/membrane that was horizontally cut and exposed to different primary antibodies.

8.1 PGRMC1, EGFR, β-TrCP, β-catenin, IκBα, and β-actin
Blot used in supplementary Figure 6a. We detected PGRMC1 and β-actin at the same membrane using the different primary antibody.

EGFR

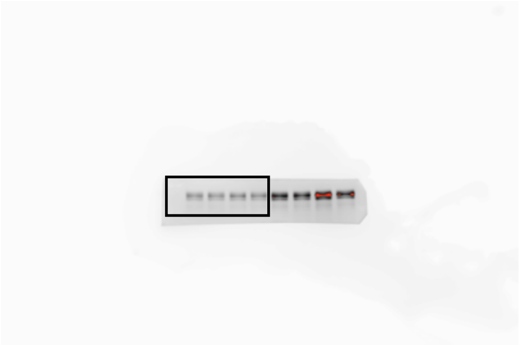

PGRMC1

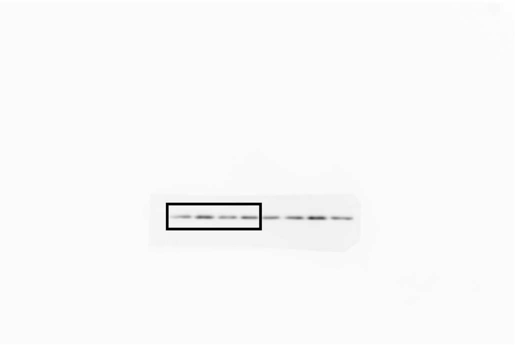

β-catenin

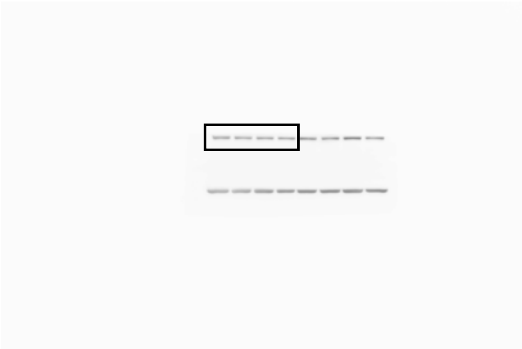

β-TrCP

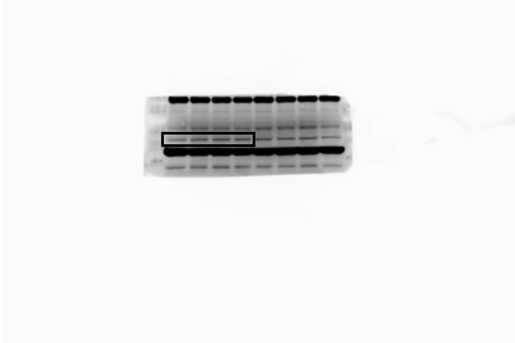

β-actin

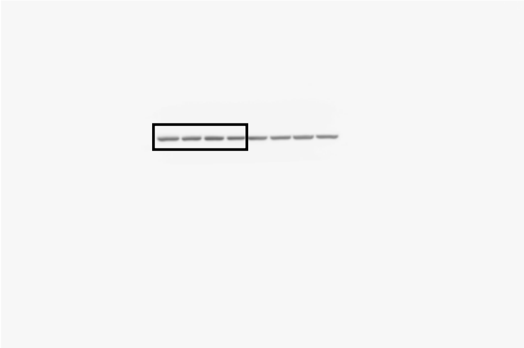

IκBα

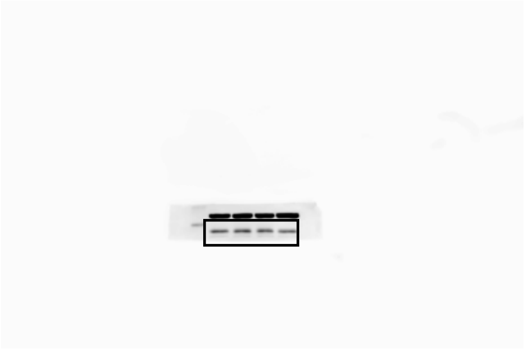

β-actin

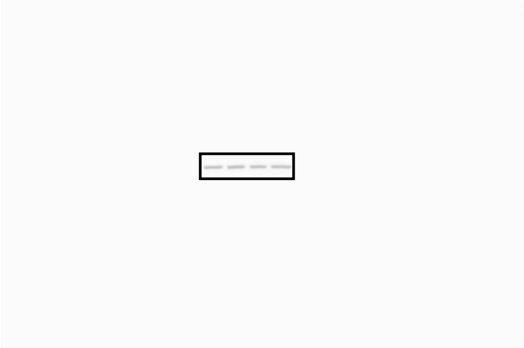

8. Original western blots cropped in Supplementary Figure 6 (Continue)

All western blots were cropped using Microsoft PowerPoint. We did not use any software to increase the contrast. The low background is a result of using 4% Block Ace for blocking solution, first, and secondary antibody. All bands from one figure origin from the same gel/membrane that was horizontally cut and exposed to different primary antibodies.

8.2 PGRMC1, EGFR, β-TrCP, β-catenin, IκBα and β-actin

Blot used in supplementary Figure 6b. We detected PGRMC1 and β-actin at the same membrane using the different primary antibody.

PGRMC1

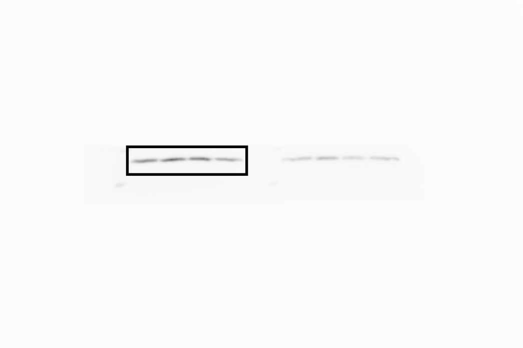

EGFR

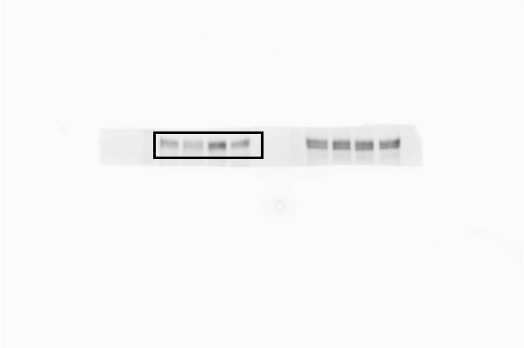

β-TrCP

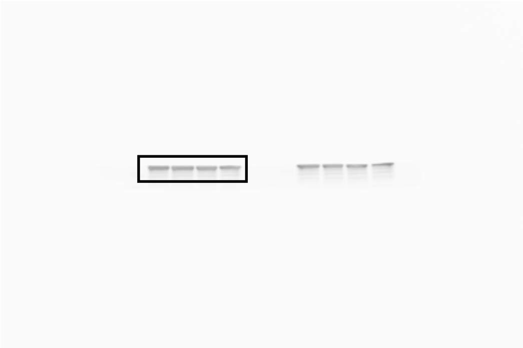

β-actin

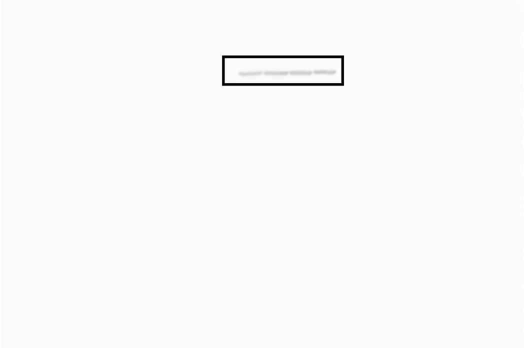

β-catenin

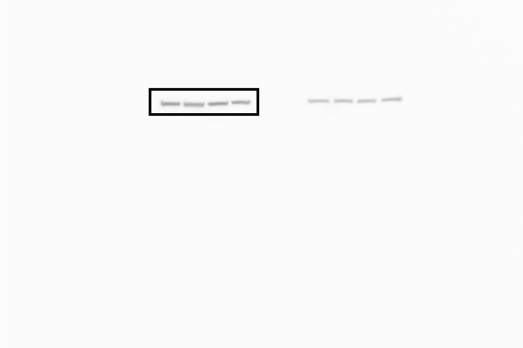

IκBα

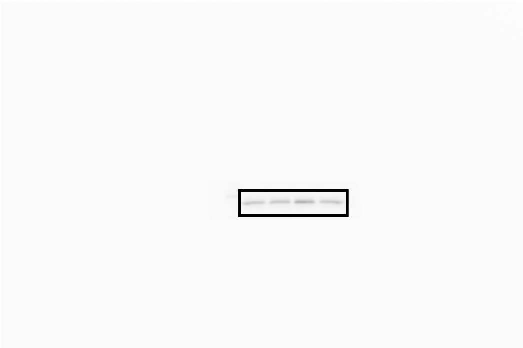

β-actin

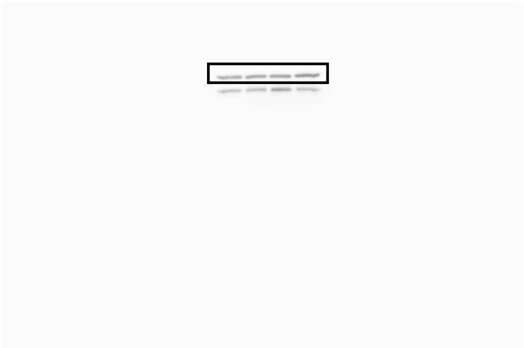

9. Original western blots cropped in Supplementary Figure 7

All western blots were cropped using Microsoft PowerPoint. We did not use any software to increase the contrast. The low background is a result of using 4% Block Ace for blocking solution, first, and secondary antibody. All bands from one figure origin from the same gel/membrane or different gel/membrane with same sample that was horizontally cut and exposed to different primary antibodies.

9.1. PGRMC1 and β-actin

Blot used in Supplementary Figure 7a. We detected PGRMC1 and β-actinat the same membrane using the different primary antibody.

PGRMC1

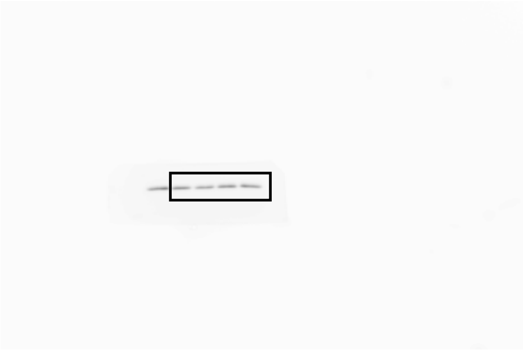

β-actin

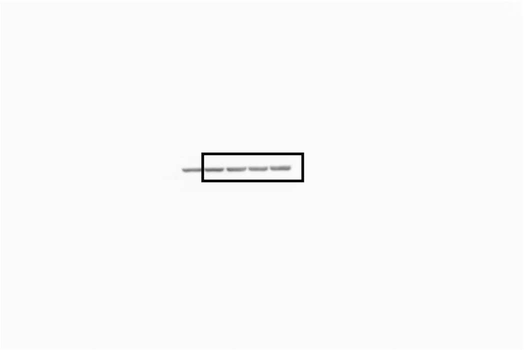

9.2. PGRMC1 and β-actin

Blot used in Supplementary Figure 7b. We detected PGRMC1 and β-actinat the same membrane using the different primary antibody.

PGRMC1

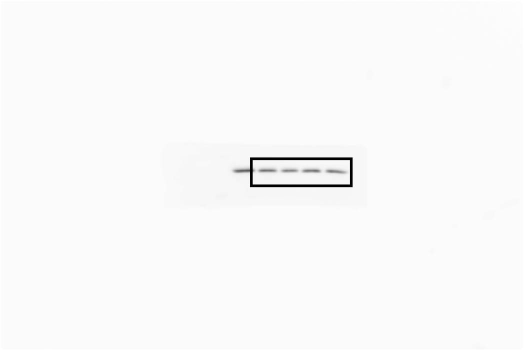

β-actin

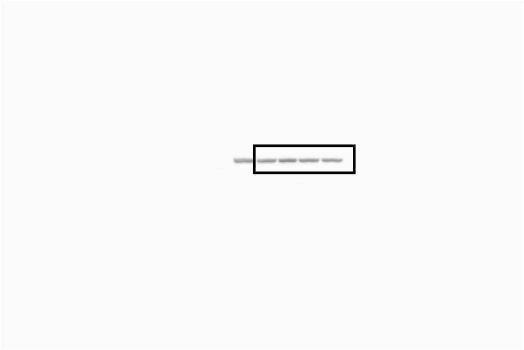

10. Original western blots cropped in Supplementary Figure 8
All western blots were cropped using Microsoft PowerPoint. We did not use any software to increase the contrast. The low background is a result of using 4% Block Ace for blocking solution, first, and secondary antibody. All bands from one figure origin from the same gel/membrane that was horizontally cut and exposed to different primary antibodies.

10.1. PGRMC1 and β-actin
Blot used in supplementary Figure 8. We detected PGRMC1 and β-actin at the same membrane using the different primary antibody.

PGRMC1

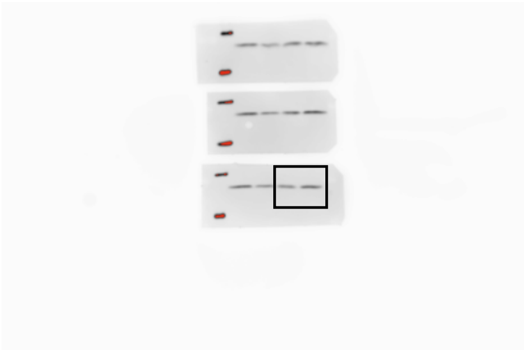

β-actin

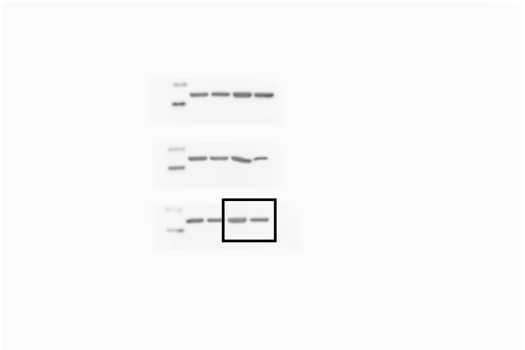

Supplement: Supplementary file 1 — Supplementary information. [file 41598_2020_61727_MOESM1_ESM.pdf]
